# Impact of the DREAMS interventions on Common mental disorders among adolescent girls and young women: Causal analysis of a prospective cohort in two Kenyan settings

**DOI:** 10.1371/journal.pmen.0000765

**Published:** 2026-11-03

**Authors:** Faith Magut, Elizabeth Kemigisha, Moses Otieno, Stephen Gakuo, Vivienne Kamire, Jane Osindo, Thomas Gachie, Sarah Mulwa, Annabelle Gourlay, Isolde Birdthistle, Daniel Grint, Daniel Kwaro, Abdhalah Ziraba, Sian Floyd

**Affiliations:** 1 Centre for Global Health Research, Kenya Medical Research Institute, Kisumu, Kenya; 2 Faculty of Epidemiology and Population Health, London School of Hygiene & Tropical Medicine, London, United Kingdom; 3 African Population and Health Research Center, Nairobi, Kenya; PLOS: Public Library of Science, UNITED KINGDOM OF GREAT BRITAIN AND NORTHERN IRELAND

## Abstract

Common mental disorders (CMD) are increasing globally among young people. The DREAMS (Determined-Resilient-Empowered-AIDS-free-Mentored-Safe) multi-component intervention package, delivered during 2016–2023, aims to reduce HIV incidence among adolescent girls and young women (AGYW). We hypothesised that social, health and economic benefits of DREAMS would reduce CMD, though this was not the program’s goal. In each of two Kenyan settings (Gem and Nairobi) a randomly selected cohort of around 1000 AGYW aged 13–22 years was enrolled during 2017–2018 and followed up during 2019–2022. CMD was measured using the Shona-Symptom-Questionnaire; a score of ≥7 was classified as `experiencing CMD’. We used inverse-probability-of-treatment-weighting to control for confounding and compared 3 groups of AGYW: those invited to DREAMS by 2018 (early invitees), during 2020–2022 (later invitees), or not invited to DREAMS (non-invitees). In 2022, the proportion experiencing CMD was 45% (463/1023) in Gem, and 46% (410/889) in Nairobi. In Gem, the estimated proportions with CMD were 49%, 45%, and 38% for the counterfactual scenarios in which all AGYW were early invitees, all were later invitees, or all were non-invitees, respectively. There was evidence of an increase in CMD prevalence when comparing early invitees with non-invitees (11%; 95% CI:2%,21%). In Nairobi, the estimated proportions with CMD were 46%, 55%, and 32% for the scenarios in which all were early invitees, all were later invitees, or all were non-invitees, respectively. There was evidence of an increase in CMD prevalence when comparing early invitees to non-invitees (14.4; 95% CI:2.1%, 26.4%) and when comparing later invitees to non-invitees (23.3%; 95% CI:5.8%,41.2%). CMD was common among AGYW in both settings. The higher CMD prevalence among DREAMS invitees compared with non-invitees might be due to increased recognition of, and willingness to disclose, symptoms of mental ill-health. Specific interventions to address CMD among AGYW should be incorporated within health and HIV prevention programmes.

## Introduction

Mental health issues are a global public health concern, and globally 20% of adolescents (aged 10–19 years) may encounter mental health problems in any given year [1]. Common mental disorders (CMD) “define a range of anxiety and depressive disorders [2], with symptoms including anxiety, depression, emotional isolation, and suicidal attempts. CMD are on the rise, and they are one of the major causes of ill health in adolescents and young people worldwide [3]. The global prevalence of CMD among adolescent girls aged 10–19 years is estimated at 31% [4]. Studies conducted in various settings, including England and various Sub-Saharan African (SSA) countries such as Tanzania, South Africa and Kenya, have reported high prevalence among AGYW ranging from 13% to 40%, respectively [5–7].

Evidence has shown that physical, emotional, and social changes, including exposure to poverty, traumatic events, or any form of violence, and lack of quality social support can make AGYW vulnerable to mental ill health [8–10]. Studies in Sub-Saharan Africa demonstrated that poverty, food insecurity, homelessness, rapid societal changes and insecurities aggravate mental distress, contributing to the susceptibility of the poor to CMD [11,12]. Other drivers of CMD among AGYW include negative life experiences such as adversities, peer pressure, disruption of family structure including orphanhood, unemployment, educational pressures, and lack of knowledge on menarche [3,13].

The consequences of CMD in AGYW include engaging in risky sexual behaviours, non-consensual sex, experience of sexual violence, education compromises, poor quality of life, and poor health-seeking behaviour [14,15]. Mental ill health also impairs personal well-being, negatively affecting the productivity of an individual and leading to poor economic outcomes [16]. In contrast, positive mental health results in resilience, coherence, negotiating ability, a positive sense of well-being, self-esteem, optimism, and healthy aging [16,17]. CMD has also been linked to HIV risk [5,18]. Evidence suggests that AGYW with CMD symptoms have a higher incidence of HIV than those without CMD symptoms [19]. This could be because CMD symptoms have an impact on sexual relationship power, unprotected sex, alcohol and substance use, and multiple partnerships.

A multi-component HIV prevention program called DREAMS Partnership (“Determined, Resilient, Empowered, AIDS-free, Mentored and Safe”) was launched in 2015 by PEPFAR (the United States President’s Emergency Plan for AIDS Relief) with the aim of reducing HIV incidence among AGYW [20]. The DREAMS package comprises interventions targeted towards individuals, wider communities, families, and AGYW partners divided into “must-have” primary interventions and secondary interventions delivered based on need (for example, condom provision for AGYW who are sexually active; post-violence care for AGYW who have experienced sexual violence) [20,21]. DREAMS did not have a specific intervention addressing mental health; however, various intervention components may have an effect on CMD (Fig 1). For example, social asset building interventions are designed to empower AGYW and increase their agency by fostering safe, supportive relationships with peers and adult mentors. These interventions enhance social capital and connectedness, both of which are likely to improve mental well-being. There is evidence that social support serves as a buffer against stress, enhances self-worth, and reduces the risk of CMD, especially in resource-constrained settings [22–24].We found evidence of an increase in social support and greater self-efficacy attributable to DREAMS in the Kenyan settings [25].

**Fig 1:**
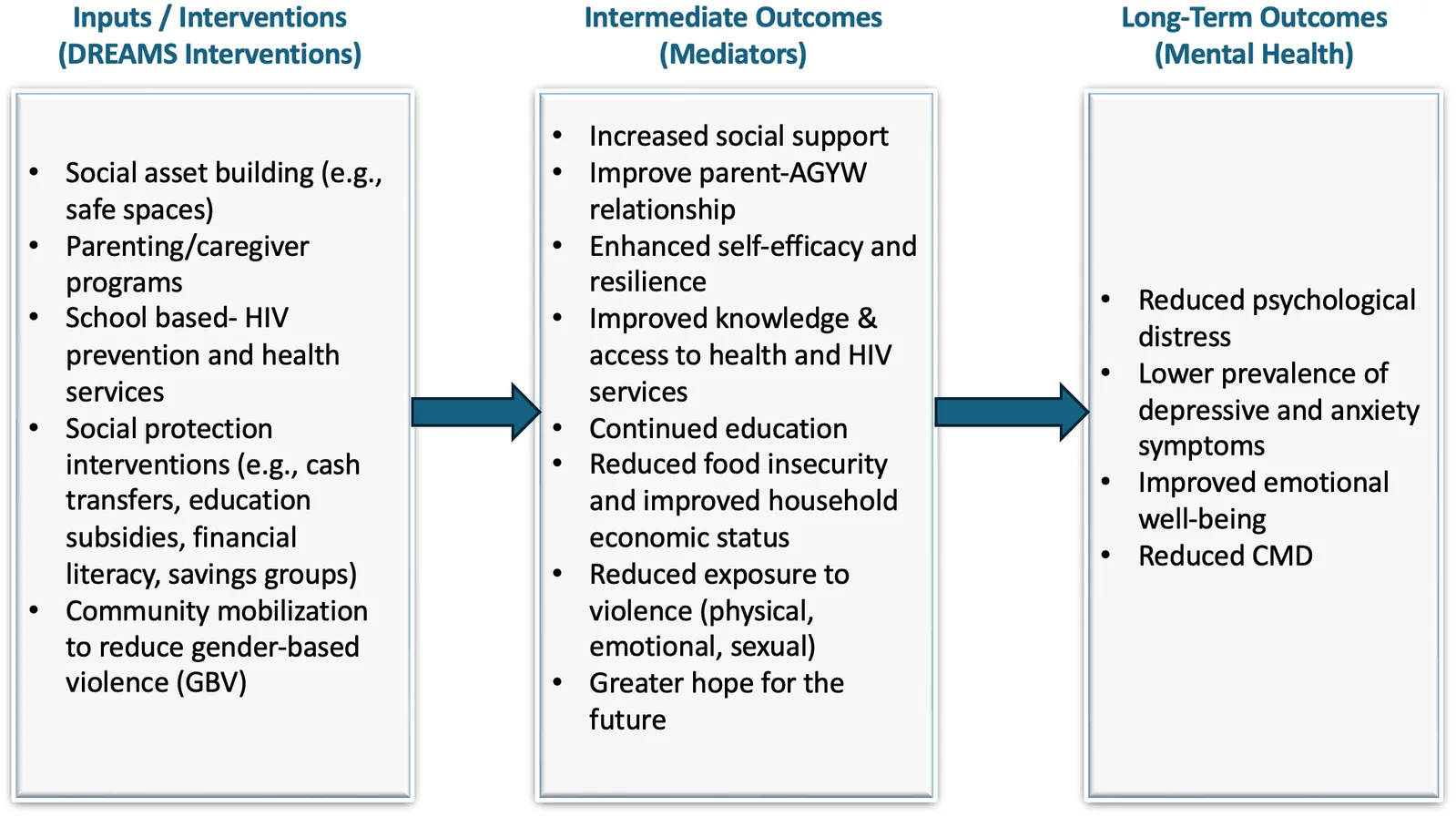
Conceptual Framework: Hypothesized Pathways from DREAMS to CMD.

DREAMS also incorporates parenting and caregiver support programs that aim to strengthen the relationships between AGYW and their caregivers. Positive family relationships characterized by trust, open communication, and emotional availability have been shown to be protective against CMD ([26]. These programs may foster environments that are emotionally nurturing and stable, contributing to lower risks of depression and anxiety among AGYW [27]. School-based HIV prevention and community mobilization interventions aim to improve knowledge, build life skills, and shift harmful community norms particularly those related to gender, sexuality, and violence. Access to sexual and reproductive health information and safer community environments can reduce fear, stigma, and internalized shame, all of which are linked to CMD. Additionally, social protection interventions such as cash transfers, vocational training, and financial literacy programs reduce economic dependency and household financial stress, which can be key sources of anxiety and psychological strain among AGYW. Economic empowerment may also contribute to continued education and a greater sense of control over life circumstances, which is associated with better mental health outcomes.

Evidence on the effect of some interventions on mental health and exposure to gaps in mental health service delivery has been demonstrated [28,29]. Studies in South Africa, Malawi, and Zimbabwe have demonstrated the benefit of interventions such as peer group clubs, self-help groups, HIV prevention programs, behaviour change interventions, cash transfers, and digital interventions in promoting well-being through awareness creation, boosting self-esteem, peer and emotional support, promoting healthy and safe relationships, and improving self-efficacy in AGYW [18,30–32]. Despite the success of these interventions and programs, the gap between demand and provision of mental health services is still an issue of concern [33]. In Kenya there are legislative issues that impede the successful implementation of its mental health policies, as well as inadequate financing and scarce facilities for dealing with mental health [34].

In this study, we aimed to estimate the prevalence of CMD among AGYW in two Kenyan settings, and to investigate whether exposure to DREAMS interventions was associated with a reduced prevalence of CMD.

## Methods

### Study design and study setting

The study was conducted in two settings, two urban informal settlements in Nairobi, and one rural setting, Gem in western Kenya. An observational closed cohort study design was employed, with a random sample of AGYW selected from a population-wide sampling frame. Gem lies within the Kenya Medical Research Institute (KEMRI) and Center for Disease Control and Prevention (CDC) Health and Demographic Surveillance System (HDSS) that was established in 2002, covering approximately 260,000 residents and 55,000 households [35]. The two sites in Nairobi lie within the Nairobi Urban and Health and Demographic Surveillance System (NUHDSS) which was also established in 2002 and covers approximately 90,000 individuals and 33,000 households [36]. The target sample size for both settings at cohort enrolment was approximately 1000. Stratified sampling was employed, seeking a balance of participants by age group: 13–17 and 18–22 years in Gem, and 15–17 and 18–22 years in Nairobi with a target sample of 500 AGYW in each age group [37].

Enrollment into the cohort study in Gem took place between January to November 2018 with a follow-up between January to November 2019. Enrolment took place in Nairobi between March to July 2017 with follow-ups between July to December 2018 and May to August 2019. The final follow-up was carried out between May 2022 to January 2023 in Gem and between September 2022 to January 2023 in Nairobi. Data were collected at the time of enrollment and during each follow-up, capturing information on household and individual characteristics such as socio-economic measures, sexual behaviour, mental health and wellbeing, invitation to participate in DREAMS, and questions relating to participation in DREAMS interventions, and a range of outcomes pertaining to biological, social, and behavioral protection. All interviews during 2017–2019 were conducted face-to-face in the households of the AGYW by trained research assistants, with electronic tablets used for data collection. In 2022, in Gem the interviews were conducted in the households of AGYW, including follow-up for AGYW who had relocated or left the study area. In Nairobi, interviews took place in a central location for those still residing in the study area, and via phone for individuals who had left the study area.

### DREAMS implementation context and DREAMS interventions

Kenya was identified as one of the priority countries for the implementation of DREAMS by the US President’s Emergency Plan for AIDS Relief (PEPFAR) [38]. The DREAMS multi-component interventions were first introduced in April 2016 in Gem and in January 2016 in Nairobi and from 2017 the interventions were being delivered together as a package [20]. The primary interventions constituted a set of seven interventions defined in 2017, that each AGYW should receive. These interventions included HIV testing services, social asset building, school-based HIV and violence prevention interventions, condom promotion and education, expanded contraceptive mix, PrEP education & provision, and financial capability training. Secondary interventions included community mobilisation and norms change, parenting and caregiver programs, post violence care for those who experienced violence as well as social protection interventions [20,39]*.* In both settings, an implementing partner that was a non-governmental organisation (NGO) was responsible for delivery and coordination of the core interventions and for facilitating referrals to health services [40]. “Safe spaces” are public places such as churches and halls and they were central to delivery of DREAMS session-based interventions in Kenya, such as social asset building and entrepreneurial and financial capability training, while school-based interventions were delivered in schools [39]. The social asset building intervention comprised of regular mentor-led meetings in “safe spaces”. This was geared towards building social capital and promoting interconnections with peers and mentors [21]. DREAMS parenting and caregiver programs aimed to improve parent-AGYW communication and relationships.

Invitation to participate in DREAMS was targeted, and thus not offered to all AGYW. The invitation criteria were based on the socio-economic and sexual risk vulnerability of the AGYW, which included pregnancy (ever or current), food insecurity, low household socioeconomic status, and being out of school or orphaned. These characteristics were used to classify the AGYW into various risk profiles and vulnerable AGYW were identified through the `Girl Roster’ census method. Implementing partners collaborated with community-based organizations, DREAMS mentors, and local leaders to prioritize enrolment for AGYW who were considered to be the most vulnerable. AGYW who were identified as vulnerable (in one or more ways) were invited to participate in DREAMS by implementing partners; they were invited through door-to-door home visits followed by enrolment interviews [40]. Invitation to participate in DREAMS occurred during 2016–2018 (“early” invitees) and again during 2020–2022 (“later” invitees), with delivery of DREAMS interventions continuing through 2023. During the COVID-19 pandemic, the delivery of DREAMS faced unique challenges. Limited access to digital technologies made remote engagement difficult for many AGYW. To address this, DREAMS programs in these regions focused on maintaining face-to-face interactions where possible, but with strict adherence to COVID-19 safety protocols and efforts were made to ensure that critical services continued with minimal disruption.

#### Measures of outcome and DREAMS intervention.

### Outcome measure - CMD

The primary outcome was Common Mental Disorders (CMD) measured in 2022 through the Shona Symptom Questionnaire (SSQ). The SSQ is a tool that screens for CMD and includes 14 questions that relate to anxiety, depression, and suicidal thoughts to obtain an overall score of individual mental health, with each question categorised as yes or no (Table A in S1 Appendix) [41]. The items are strong indicators of CMD [42]. The main study that developed and validated the SSQ tool was conducted in Zimbabwe among a random sample of primary care attendees aged ≥16 years, and indicated a good internal consistency (Cronbach alpha = 0.85) and suggested that a cut-off of 7 or 8 (out of a maximum score of 14) was satisfactory to define experience CMD with a discriminatory ability of 0.88 [42]. Another study in Zimbabwe which validated the SSQ on adolescents and young people established that a cut-off of 5 and above was suitable [41].

In our study, 13 items similar to the 14 SSQ items were available, and they were used to generate the SSQ score. One question, “Was your stomach aching in the past week?,” was excluded as it does not explicitly measure mental well-being. The 13 items (each categorised as 0 or 1) were summed to determine the total score, ranging from 0 to 13 where 0 indicates a low score for CMD, i.e., good mental health, and 13 indicates a high score for CMD, i.e., poor mental health.

We derived a binary outcome from the mental health scores. AGYW with a score of ≥7 were categorized as “experiencing CMD,” while those with scores <7 were classified as “not experiencing CMD”, based on previous validation of the SSQ [42]. However, this measure does not reflect a definitive diagnosis of CMD. To contribute to the robustness of our findings, we conducted a sensitivity analysis where we defined the cut-off for CMD using a more stringent value of 10.

### DREAMS intervention measure

We compared AGYW who self-reported they were, or were not, invited to participate in DREAMS. Invitation to participate in DREAMS during 2018–2022, was classified into three categories. Early invitees were AGYW who were invited during 2016–2018, later invitees were AGYW who were invited during 2020–2022, and non-invitees were AGYW who were not invited to DREAMS by the time of follow-up in 2022. Invitation to DREAMS (yes or no) was used as our measure of exposure to DREAMS interventions, irrespective of participation in DREAMS interventions and the number of interventions that were actually received, to provide a measure of programme effectiveness and one that corresponded to an “intention-to-treat” analysis. Among invitees, participation in DREAMS interventions was high with around 70% receiving  ≥ 3 primary package interventions by 2019 [39].

AGYW who were not invited to participate in the DREAMS were not excluded from accessing other services available in the community. These may have included standard health, social, or educational services provided outside of the DREAMS programme.

#### Confounding variables.

We constructed a Directed Acyclic Graph (DAG) to conceptualise the causal relationship between invitation to DREAMS and CMD using Dagitty [43]. The DAG was used to identify confounding variables that needed to be adjusted for to estimate the causal effect of DREAMS on CMD [44]. It was essential to appropriately identify confounders for the validity of the conditional exchangeability (no residual confounding) assumption [45]. Identification of these factors was based on expert knowledge about DREAMS implementation and related literature. Age was conceptualized to influence education, pregnancy, sexual history, socio-economic status (SES), food insecurity, and access to credit, given that older AGYW are more likely to have sexual experience, earn income, and access finances, while younger AGYW are more likely to still be in school. Parental education was considered to shape SES, food insecurity, and access to credit through increased knowledge and earning, which in turn support educational attainment. Household SES was hypothesized to influence education by determining the ability to afford school fees and materials. Orphanhood was considered to affect education, SES, and food insecurity through the loss of parental support, while also increasing vulnerability to early pregnancy and risky sexual behaviours. Pregnancy and sexual history were further hypothesized to affect education through increased likelihood of school drop-out. Following this, age, highest level of education, socio-economic status, food security, marital status, pregnancy and sexual history, orphanhood status, and schooling status (in or out of school) were identified as potential confounders.

These factors represent socio-economic and sexual risk vulnerability characteristics that informed eligibility and invitation to DREAMS and are plausibly associated with both the exposure (invitation to DREAMS) and the outcome (CMD). For example, AGYW who were orphaned, had experienced pregnancy, or were from households with low socioeconomic status were prioritised for invitation, reflecting targeted programme delivery to higher-risk groups. Additionally, we adjusted for the highest education level of the household head, experience of violence in the previous 12 months, experience of household shocks in the previous 12 months, and self-efficacy, as they were hypothesized to be determinants of CMD (Fig 2).

**Fig 2:**
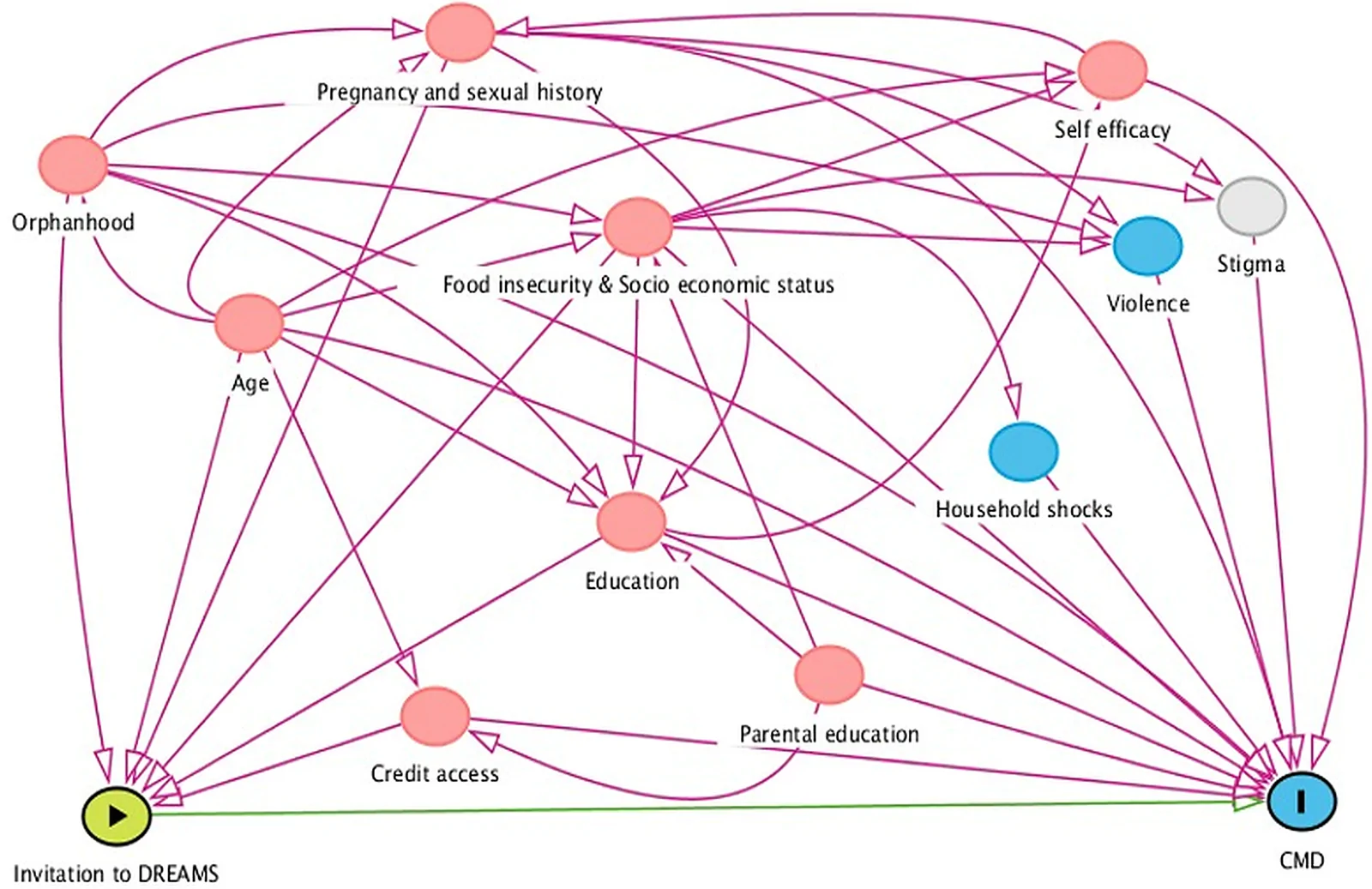
Directed Acyclic graph of the association between Invitation to DREAMS and the outcome of CMD among AGYW in two settings in Kenya.

#### Statistical analysis.

First, we summarised the proportion of AGYW who were followed up in 2022, overall and according to characteristics measured at cohort enrolment. We then summarised the proportion of AGYW who experienced CMD in 2022, stratified by age group (13–17 and 18–22 years) at cohort enrolment in 2017/2018 and by invitation to participate in DREAMS during 2016–2022. Among these AGYW who were followed up in 2022, we compared early invitees, later invitees and non-invitees by key socio-demographic and socio-economic characteristics at the time of enrolment, and we also compared later invitees and non-invitees for these characteristics based on 2019 follow up data.

Next, using a causal inference framework, we estimated the causal effect of DREAMS on CMD by comparing three counterfactual scenarios in which all AGYW were early invitees, all were later invitees, and all were non-invitees. Our primary estimand is the population average treatment effect (ATE) of invitation to DREAMS on CMD, defined as the contrast in the proportion of AGYW with CMD under hypothetical intervention strategies in which all AGYW were invited to participate in DREAMS early (2016–2018), or all were invited later (2020–2022), compared with a scenario in which no AGYW were invited to participate in DREAMS.

Our primary analysis approach for comparing these 3 counterfactual scenarios was through inverse-probability-of-treatment-weighting (IPTW), with the probability of treatment estimated using propensity score models [46]. Using IPTW enabled time-varying confounders – characteristics that were determinants of whether an AGYW was invited to DREAMS but also might be influenced by participation in DREAMS – to be accounted for, in the context that AGYW were invited to participate in DREAMS during 2016–2018 (“early invitees”) and again during 2020–2022 (“later invitees”). To account for this change over time in whether an AGYW was an invitee or remained a non-invitee, the probability-of-treatment was estimated in a two-step process using two propensity score models. The first step was to fit a propensity score model to estimate the probability of being an early invitee. This was done using a logistic regression model in which invitation to DREAMS by the time of the 2018 interview was specified as the ‘outcome’ (yes or no), and explanatory variables were measured at cohort enrolment (2017 in Nairobi and 2018 in Gem); the explanatory variables were confounders identified in the DAGs plus independent determinants of CMD. The second step was to fit a propensity score model to estimate the probability of being a later invitee, among AGYW who had not been invited to DREAMS by 2018. This was done using a logistic regression model in which invitation to DREAMS during 2020–2022 was specified as the ‘outcome’ (yes or no), and explanatory variables were measured in 2019 (or 2018, if the AGYW was not followed up in 2019), apart from age for which (as with propensity score model 1) we used age group at enrolment. As with the first propensity score model, explanatory variables were confounders identified in the DAGs plus independent determinants of CMD. We assessed the “positivity assumption” [47] by examining the distribution of each of the two propensity scores using histograms and kernel density plots, comparing the overlap in the propensity scores between early invitees versus AGYW who were not early invitees (propensity score model 1), and between later invitees and non-invitees (propensity score model 2) (Figure A in S1 Appendix and Figure B in S1 Appendix). Unbiased estimates of the causal effects can be obtained (through IPTW) if the overlap is good [48]. In the Nairobi setting, almost all AGYW who had a propensity score to be an early invitee that was < 0.20 were non-invitees; because of this lack of overlap, for the Nairobi setting we excluded AGYW who had a propensity score of <0.2 from the primary analysis.

We then used the two propensity scores (estimated probabilities of being invited to DREAMS) to estimate the inverse-probability-of-treatment weights. The inverse-probability-of-treatment weights for the three comparison groups were as follows; $\frac{\text{1}}{\pi_{\text{1}}}$ for early invitees, $\frac{\text{1}}{\text{1}-\pi_{\text{1}}}*\frac{\text{1}}{\pi_{\text{2}}}$, for the later invitees, and $\frac{\text{1}}{\text{1}-\pi_{\text{1}}}*\frac{\text{1}}{\text{1}-\pi_{\text{2}}}$ for non-invitees, where $\pi_{\text{1}}$ represents the estimated probability (propensity) of being an early invitee and $\pi_{\text{2}}$ represents the estimated probability (propensity) of being a later invitee among AGYW who were not early invitees.

When using the IPTW approach to analysis, individuals with estimated “probabilities of treatment” that are close to 0 are assigned a large weight and they become more influential than others. Extreme weights affect the estimated effects by inflating the variance and confidence intervals. Thus, extreme weights can be handled through either weight stabilization or weight truncation [49]. In this study, we used stabilized inverse-probability-of-treatment weights. Stabilizing the weights was achieved by replacing the numerator of the weight with the overall probability of an AGYW being in that category of “treatment”. The respective stabilized weights for the three comparison groups were as follows: $\frac{P(T=\text{1})}{\pi_{\text{1}}}$ for the early invitees, $\frac{\text{1}-P(T=\text{1})}{\text{1}-\pi_{\text{1}}}*\frac{P(T=\text{2})}{\pi_{\text{2}}}$ for the later invitees and $\frac{\text{1}-P(T=\text{1})}{\text{1}-\pi_{\text{1}}}*\frac{\text{1}-P(T=\text{2})}{\text{1}-\pi_{\text{2}}}$ for the non-invitees, where P(T = 1) was the overall probability of being an early invitee and P(T = 2) was the overall probability of being a later invitee among AGYW who were not early invitees.

To account for loss to follow-up in 2022, we used the inverse-probability-of-censoring weights (IPCW) approach, where those AGYW who were followed up in 2022 were weighted according to their estimated probability of being followed up. The probability of being followed up was estimated using a logistic regression model in which follow-up in 2022 was specified as the ‘outcome’ (yes or no), and characteristics measured at enrolment (including age, invitation to DREAMS by 2018, pregnancy and sexual history) were considered as explanatory variables. Characteristics that were strongly associated with follow-up were selected. The IPCW approach assumes that, conditional on these characteristics included in the censoring model, loss to follow-up is independent of the potential CMD outcomes.

The IPCW for each AGYW was calculated by taking the inverse of their estimated probability of being followed up. The final weight for the IPTW analysis was constructed by multiplying together the stabilized weight (as described above) and the weight obtained from the probability of being followed up. To assess extreme weights, we examined the distribution of the stabilized weights using histograms (Figure C in S1 Appendix and Figure D in S1 Appendix).Next, we fitted a linear regression model with CMD in 2022 as the outcome variable (yes or no) and invitation to DREAMS as the (only) explanatory variable, with each AGYW weighted as described above. The average causal effect (ACE) was the coefficient of the explanatory variable (invitation to DREAMS, in 3 categories) in this model. The ACE was determined both for the overall sample and stratified by younger and older (at the time of cohort enrolment) age groups. The age-specific estimates were obtained using the same linear regression model but restricting the analysis to the age group of interest. A bootstrap method with 1000 replicates was used to estimate the 95% confidence intervals of the estimates.

#### Sensitivity analysis.

To examine the robustness of our estimate of the impact of DREAMS on CMD obtained from the IPTW analysis, we conducted two alternative analyses for comparison. First, we conducted IPTW truncation, where the stabilized weights with a value >3 were truncated. Secondly, we conducted a sensitivity analysis of the binary CMD outcome using a more stringent cut-off of ≥10 on the SSQ score.

#### Assessing conditional exchangeability (residual confounding), in the IPTW analysis.

We assessed the covariate balance that was achieved from using the inverse-probability-of-treatment weighting (IPTW) approach. Standardized mean differences (SMD) of the covariates (that were included in the propensity score models) were employed to evaluate covariate balance (comparing early invitees, later invitees, and non-invitees) both before IPTW and after the re-weighting process (Figure E in S1 Appendix, Figure F in S1 Appendix, Figure G in S1 Appendix and Figure H in S1 Appendix).

#### Ethical considerations.

Ethical approvals for the study were obtained from the research ethics committees at the London School of Hygiene and Tropical Medicine (LSHTM), Amref Health Africa, and the Kenyan Medical Research Institute (KEMRI). Informed written consent was acquired from participants aged 18 years or older. For legal minors under 18 years of age, consent was obtained from a legal guardian, and assent was sought from the adolescent.

## Results

### Enrolment profile and retention

Tables 1 and 2 present cohort follow-up in 2022, based on socio-demographic and socio-economic characteristics at enrolment. In Gem, 88% of 1171 AGYW enrolled in 2018 were followed up in 2022: 92% among those aged 13–17 years and 82% among those aged 18–22 years at enrolment. In Nairobi, 82% of 1081 AGYW were followed up in 2022; 87% among those aged 15–17 years and 77% among those aged 18–22 years at enrolment. Retention was higher among DREAMS early invitees than non-invitees, in both settings (e.g., 91% vs. 83% in Gem, 87% vs. 73% in Nairobi) and among both younger and older AGYW. Retention was also higher for AGYW who, at the time of cohort enrolment, had never had sex compared to those who had ever been pregnant (e.g., 92% vs. 75% in Gem, 88% vs. 73% in Nairobi). Differences in retention were smaller for household socio-economic position, food security, orphanhood, and educational level (Tables 1 and 2).

**Table 1:** Cohort retention in 2022, by characteristics at enrolment (in 2018) in Gem (N = 1171).

	Overall		13-17 years		18-22 years	
	*n/N*	*%*	*n/N*	*%*	*n/N*	*%*
**Overall**	1027/1171	87.7	628/684	91.8	399/487	81.9
**Invited to participate in DREAMS by 2018**						
No	427/514	83.1	252/285	88.4	175/229	76.4
Yes	600/657	91.3	376/399	94.2	224/258	86.8
**Educational attainment**						
Primary/None	440/481	91.5	362/386	93.8	78/95	82.1
Secondary and above	359/411	87.3	138/151	91.4	221/260	85
Unknown	228/279	81.7	128/147	87.1	100/132	75.8
**SES and food insecurity**						
Food insecure, low medium high SES	227/253	89.7	129/136	94.9	98/117	83.8
Food secure, low SES	225/262	85.9	144/156	92.3	81/106	76.4
Food secure, medium SES	189/210	90	120/131	91.6	69/79	87.3
Food secure, high SES	386/446	86.5	235/261	90.0	151/185	81.6
**Self-assessed poverty of household**						
Very Poor	137/151	90.7	97/100	97.0	40/51	78.4
Moderately poor	734/844	87	428/474	90.3	306/370	82.7
Not Poor	156/176	88.6	103/110	93.6	53/66	80.3
**Orphanhood status**						
No	623/687	90.7	395/426	92.7	228/261	87.4
Maternal	36/40	90.0	18/20	90.0	18/20	90.0
Paternal	96/107	89.7	53/55	96.4	43/52	82.7
Double orphan	32/39	82.1	15/15	100	17/24	70.8
Unknown	240/298	80.5	147/168	87.5	93/130	71.5
**Access to credit**						
No	517/580	89.1	289/308	93.8	228/272	83.8
Yes	320/367	87.2	182/198	91.9	138/169	81.7
Don’t know’	190/224	84.8	157/178	88.2	33/46	71.7
**Ever had sex, ever pregnant composite variable**						
Never had sex	705/766	92.0	558/600	93.0	147/166	88.6
Ever sex, never pregnant	161/190	84.7	55/61	90.2	106/129	82.2
Ever pregnant	161/215	74.9	15/23	65.2	146/192	76
**Highest education of the head of the household**						
No schooling/incomplete primary	212/245	86.5	137/147	93.2	75/98	76.5
Complete primary	238/263	90.5	120/133	90.2	118/130	90.8
Incomplete/complete secondary	225/269	83.6	127/139	91.4	98/130	75.4
Tertiary	88/101	87.1	45/49	91.8	43/52	82.7
Don’t know	264/293	90.1	199/216	92.1	65/77	84.4

**Table 2:** Cohort retention in 2022 by characteristics at enrolment (in 2017) in Nairobi (N = 1081).

	Overall		15-17 years		18-22 years	
	n/N	%	n/N	%	n/N	%
**Overall**	889/1081	82.2	477/547	87.2	412/534	77.2
**Invited to participate in DREAMS by 2018**						
No	253/347	72.9	99/124	79.8	154/223	69.1
Yes	636/734	86.6	378/423	89.4	258/311	83
**DSS study site**						
Korogocho	515/617	83.5	277/317	87.4	238/300	79.3
Viwandani	374/464	80.6	200/230	87	174/234	74.4
**Currently in school**						
No	338/455	74.3	70/88	79.5	268/367	73
Yes	551/626	88	407/459	88.7	144/167	86.2
**Highest education level completed**						
None/incomplete primary	105/124	84.7	72/82	87.8	33/42	78.6
Complete primary	167/217	77	99/119	83.2	68/98	69.4
Some secondary	416/491	84.7	287/325	88.3	129/166	77.7
Complete secondary/Tertiary	201/249	80.7	19/21	90.5	182/228	79.8
**Food insecurity**						
No	593/730	81.2	313/362	86.5	280/368	76.1
Yes	296/351	84.3	164/185	88.6	132/166	79.5
**Wealth tertile**						
Poor	299/361	82.8	165/191	86.4	134/170	78.8
Medium	297/360	82.5	168/196	85.7	129/164	78.7
Wealthy	293/360	81.4	144/160	90	149/200	74.5
**Self-assessed household poverty**						
Very poor	115/138	83.3	56/65	86.2	59/73	80.8
Moderately poor	703/859	81.8	380/436	87.2	323/423	76.4
Not poor	71/84	84.5	41/46	89.1	30/38	78.9
**Orphanhood status**						
Not an orphan	656/744	88.2	375/417	89.9	281/327	85.9
Single/double orphan	170/209	81.3	83/99	83.8	87/110	79.1
Unknown/missing	63/128	49.2	19/31	61.3	44/97	45.4
**Access to credit**						
No	449/550	81.6	230/270	85.2	219/280	78.2
Yes	395/482	82.0	219/247	88.7	176/235	74.9
Don’t know	45/49	91.8	28/30	93.3	17/19	89.5
**Ever had sex, ever pregnant composite variable**						
Never had sex	564/644	87.6	419/481	87.1	145/163	89.0
Ever sex, never pregnant	108/138	78.3	32/35	91.4	76/103	73.8
Ever pregnant	217/299	72.6	26/31	83.9	191/268	71.3
**Marital status**						
never married	728/843	86.4	466/534	87.3	262/309	84.8
Prev/current married/living w partner	161/238	67.6	11/13	84.6	150/225	66.7
**Is the AGYW the household head**						
No	850/1031	82.4	470/540	87.0	380/491	77.4
Yes	39/50	78.0	7/7	100	32/43	74.4
**Highest education of the head of the household**						
No schooling/incomplete primary	217/267	81.3	127/149	85.2	90/118	76.3
Complete primary	296/374	79.1	138/157	87.9	158/217	72.8
Incomplete/complete secondary	58/72	80.6	22/30	73.3	36/42	85.7
Tertiary	116/131	88.5	74/79	93.7	42/52	80.8
Don’t know	202/237	85.2	116/132	87.9	86/105	81.9

### Characteristics of non-invitees, early invitees, and later invitees, at cohort enrolment

In Gem, among AGYW followed up in 2022, 15% (156/1023) were non invitees, 59% (599/1023) were early invitees and 26% (268/1023) were later invitees. Both early and later invitees were younger than non-invitees, with 63% of early invitees and 66% of later invitees aged 13–17 years at cohort enrolment compared with 47% of non-invitees. A higher percentage of early and later invitees had none or only primary education at enrolment, at 45% and 43%, respectively compared to 33% of non-invitees, and a higher percentage of early and later invitees were food insecure at enrolment, 25% and 18% respectively compared to 16% among non-invitees (Table 3).

**Table 3:** Characteristics of AGYW at enrolment by age and invitation to DREAMS in Gem (N = 1023).

		Age		Overall		
		Age category (years)		Invited to DREAMS		
	Overall, N = 1023	13-17, N = 627	18-22, N = 396	Non-invitees, N = 156	Early invitees, N = 599	Later invitees, N = 268
	N(%)	N(%)	N(%)	N(%)	N(%)	N(%)
**Age group (years)**						
13-17	627(61.3)	627(100.0)	0(0.0)	73(46.8)	376(62.8)	178(66.4)
18-22	396(38.7)	0(0.0)	396(100.0)	83(53.2)	223(37.2)	90(33.6)
**Educational attainment**						
Primary/None	439(42.9)	362(57.7)	77(19.4)	52(33.3)	271(45.2)	116(43.3)
Secondary and above	358(35.0)	138(22.0)	220(55.6)	63(40.4)	220(36.7)	75(28.0)
Unknown	226(22.1)	127(20.3)	99(25.0)	41(26.3)	108(18.0)	77(28.7)
**SES and food insecurity**						
Food insecure, low medium high SES	225(22.0)	128(20.4)	97(24.5)	25(16.0)	152(25.4)	48(17.9)
Food secure, low SES	224(21.9)	144(23.0)	80(20.2)	27(17.3)	142(23.7)	55(20.5)
Food secure, medium SES	189(18.5)	120(19.1)	69(17.4)	29(18.6)	111(18.5)	49(18.3)
Food secure, high SES	385(37.6)	235(37.5)	150(37.9)	75(48.1)	194(32.4)	116(43.3)
**Self-assessed household poverty**						
Very Poor	137(13.4)	97(15.5)	40(10.1)	17(10.9)	85(14.2)	35(13.1)
Moderately poor	732(71.6)	428(68.3)	304(76.8)	100(64.1)	439(73.3)	193(72.0)
Not Poor	154(15.1)	102(16.3)	52(13.1)	39(25.0)	75(12.5)	40(14.9)
**Orphanhood status**						
No	620(60.6)	395(63.0)	225(56.8)	97(62.2)	364(60.8)	159(59.3)
Maternal	36(3.5)	18(2.9)	18(4.5)	7(4.5)	21(3.5)	8(3.0)
Paternal	96(9.4)	53(8.5)	43(10.9)	13(8.3)	58(9.7)	25(9.3)
Double orphan	32(3.1)	15(2.4)	17(4.3)	10(6.4)	15(2.5)	7(2.6)
Unknown	239(23.4)	146(23.3)	93(23.5)	29(18.6)	141(23.5)	69(25.7)
**Ever had sex, ever pregnant composite variable**						
Never had sex	703(68.7)	557(88.8)	146(36.9)	93(59.6)	433(72.3)	177(66.0)
Ever sex, never pregnant	161(15.7)	55(8.8)	106(26.8)	27(17.3)	82(13.7)	52(19.4)
Ever pregnant	159(15.5)	15(2.4)	144(36.4)	36(23.1)	84(14.0)	39(14.6)
**Access to credit**						
No	515(50.3)	288(45.9)	227(57.3)	85(54.5)	294(49.1)	136(50.7)
Yes	318(31.1)	182(29.0)	136(34.3)	50(32.1)	193(32.2)	75(28.0)
Don’t know	190(18.6)	157(25.0)	33(8.3)	21(13.5)	112(18.7)	57(21.3)
**Highest education of the head of the household**						
No schooling/incomplete primary	212(20.7)	137(21.9)	75(18.9)	28(17.9)	129(21.5)	55(20.5)
Complete primary	237(23.2)	119(19.0)	118(29.8)	33(21.2)	146(24.4)	58(21.6)
Incomplete/complete secondary	224(21.9)	127(20.3)	97(24.5)	36(23.1)	132(22.0)	56(20.9)
Tertiary	88(8.6)	45(7.2)	43(10.9)	20(12.8)	45(7.5)	23(8.6)
Don’t know	262(25.6)	199(31.7)	63(15.9)	39(25.0)	147(24.5)	76(28.4)

In Nairobi, by 2022 14% (121/889) of the AGYW were non invitees, 71% (636/889) were early invitees and 15% (132/889) were later invitees. Both early and later invitees were younger than non-invitees, with 59% of early invitees and 45% of later invitees aged 15–17 years at enrolment compared to 32% of non-invitees. A higher percentage of the early and later invitees were in school at enrolment, at 68% and 54% respectively, compared to 40% for non-invitees, and early and later invitees were more likely to live in households with lower socio-economic position, 35% and 34% respectively, compared to 25% among non-invitees (Table 4).

**Table 4:** Characteristics of AGYW at enrolment by age and invitation to DREAMS in Nairobi (N = 889).

		Age		Overall		
		Age category (years)		Invited to DREAMS		
	Overall N = 889	15-17 N = 477	18-22 N = 412	Non-invitees, N = 121	Early Invitees, N = 636	Later Invitees, N = 132
	N(%)	N(%)	N(%)	N(%)	N(%)	N(%)
**Age group (years)**						
15-17	477(53.7)	477(53.7)	0(0.0)	39(32.2)	378(59.4)	60(45.4)
18-22	412(46.3)	0(0.0)	412(46.3)	82(67.8)	258(40.4)	72(54.6)
**DSS study site**						
Korogocho	515(57.9)	277(58.1)	238(57.8)	61(50.4)	365(57.4)	89(67.4)
Viwandani	374(42.1)	200(41.9)	174(42.2)	60(49.6)	271(42.6)	43(32.6)
**Currently in school**						
No	338(38.0)	70(14.7)	268(65.0)	73(60.3)	204(32.1)	61(46.2)
Yes	551(62.0)	407(85.3)	144(35.0)	48(39.7)	432(67.9)	71(53.8)
**Highest education level completed**						
None/incomplete primary	105(11.8)	72(15.1)	33(8.0)	18(14.9)	70(11.0)	17(12.9)
Complete primary	167(18.8)	99(20.8)	68(16.5)	29(24.0)	110(17.3)	28(21.2)
Some secondary	416(46.8)	287(60.2)	129(31.3)	28(23.1)	337(53.0)	51(38.6)
Complete secondary/Tertiary	201(22.6)	19(4.0)	182(44.2)	46(38.0)	119(18.7)	36(27.3)
**Food insecurity**						
No	593(66.7)	313(65.6)	280(68.0)	89(73.6)	406(63.8)	98(74.2)
Yes	296(33.3)	164(34.4)	132(32.0)	32(26.4)	230(36.2)	34(25.8)
**Wealth tertile**						
Poor	299(33.6)	165(34.6)	134(32.5)	30(24.8)	224(35.2)	45(34.1)
Medium	297(33.4)	168(35.2)	129(31.3)	49(40.5)	201(31.6)	47(35.6)
Wealthy	293(33.0)	144(30.2)	149(36.2)	42(34.7)	211(33.2)	40(30.3)
**Self-assessed household poverty**						
Very poor	115(12.9)	56(11.7)	59(14.3)	12(9.9)	89(14.0)	14(10.6)
Moderately poor	703(79.1)	380(79.7)	323(78.4)	100(82.6)	503(79.1)	100(75.8)
**Orphanhood status**						
Not an orphan	656(73.8)	375(78.6)	281(68.2)	68(56.2)	499(78.5)	89(67.4)
Single/double orphan	170(19.1)	83(17.4)	87(21.1)	31(25.6)	116(18.2)	23(17.4)
Unknown/missing	63(7.1)	19(4.0)	44(10.7)	22(18.2)	21(3.3)	20(15.2)
**Access to credit**						
No	449(50.5)	230(48.2)	219(53.2)	65(53.7)	325(51.1)	59(44.7)
Yes	395(44.4)	219(45.9)	176(42.7)	51(42.2)	277(43.6)	67(50.8)
Don’t know	45(5.1)	28(5.9)	17(4.1)	5(4.1)	34(5.4)	6(4.6)
**Ever had sex, ever pregnant composite variable**						
Never had sex	564(63.4)	419(87.8)	145(35.2)	54(44.6)	439(69.0)	71(53.8)
Ever sex, never pregnant	108(12.1)	32(6.7)	76(18.4)	20(16.5)	67(10.5)	21(15.9)
Ever pregnant	217(24.4)	26(5.5)	191(46.4)	47(38.8)	130(20.4)	40(30.3)
**Marital status**						
never married	728(81.9)	466(97.7)	262(63.6)	78(64.5)	548(86.2)	102(77.3)
Prev/currently married/living w partner	161(18.1)	11(2.3)	150(36.4)	43(35.5)	88(13.8)	30(22.7)
**Is the AGYW the Household Head**						
No	850(95.6)	470(98.5)	380(92.2)	110(90.9)	616(96.9)	124(93.9)
Yes	39(4.4)	7(1.5)	32(7.8)	11(9.1)	20(3.1)	8(6.1)
**Highest education of the head of the household**						
No schooling/incomplete primary	202(22.7)	116(24.3)	86(20.9)	21(17.4)	160(25.2)	21(15.9)
Complete primary	217(24.4)	127(26.6)	90(21.8)	19(15.7)	170(26.7)	28(21.2)
Incomplete/complete secondary	296(33.3)	138(28.9)	158(38.3)	53(43.8)	197(31.0)	46(34.9)
Tertiary	58(6.5)	22(4.6)	36(8.7)	9(7.4)	35(5.5)	14(10.6)
Don’t know	116(13.0)	74(15.5)	42(10.2)	19(15.7)	74(11.6)	23(17.4)

### CMD prevalence among AGYW in 2022, by age and invitation to DREAMS

In both Gem and Nairobi, approximately 45% of AGYW experienced CMD in 2022. CMD was more prevalent among older AGYW compared to younger AGYW. Among the older group, 54% in Gem and 50% in Nairobi experienced CMD, while the younger age group had slightly lower percentages, with 40% in Gem and 43% in Nairobi (Table 5). CMD was more prevalent among both early and later invitees when compared to non-invitees (Table 5 and Fig 3). Among the early invitees, 45% in Gem and 47% in Nairobi experienced CMD, while among the later invitees, 44% in Gem and 52% in Nairobi experienced CMD. In contrast, non-invitees had a lower CMD prevalence, with 37% in Gem and 39% in Nairobi (Table 5).

**Table 5:** CMD prevalence in Gem and Nairobi in 2022, overall and by age group at cohort enrolment and DREAMS invitation status.

	Overall		Non-invitees		Early invitees		Later invitees	
	n/N	%	n/N	%	n/N	%	n/N	%
**Gem**								
**CMD** (SSQ ≥ 7)								
Overall (total)	463/1023	**45.3**	58/156	**37.2**	287/599	**47.9**	118/268	**44.0**
13-17 years	253/627	**40.4**	22/73	**30.1**	162/376	**43.1**	69/178	**38.8**
18-22 years	210/396	**53.0**	36/83	**43.4**	125/223	**56.1**	49/90	**54.4**
**Nairobi**								
**CMD** (SSQ ≥7)								
Overall (total)	410/889	**46.1**	47/121	**38.8**	295/636	**46.4**	68/132	**51.5**
15-17 years	203/477	**42.6**	10/39	**25.6**	165/378	**43.7**	28/60	**46.7**
18-22 years	207/412	**50.2**	37/82	**45.1**	130/258	**50.4**	40/72	**55.6**

**Fig 3:**
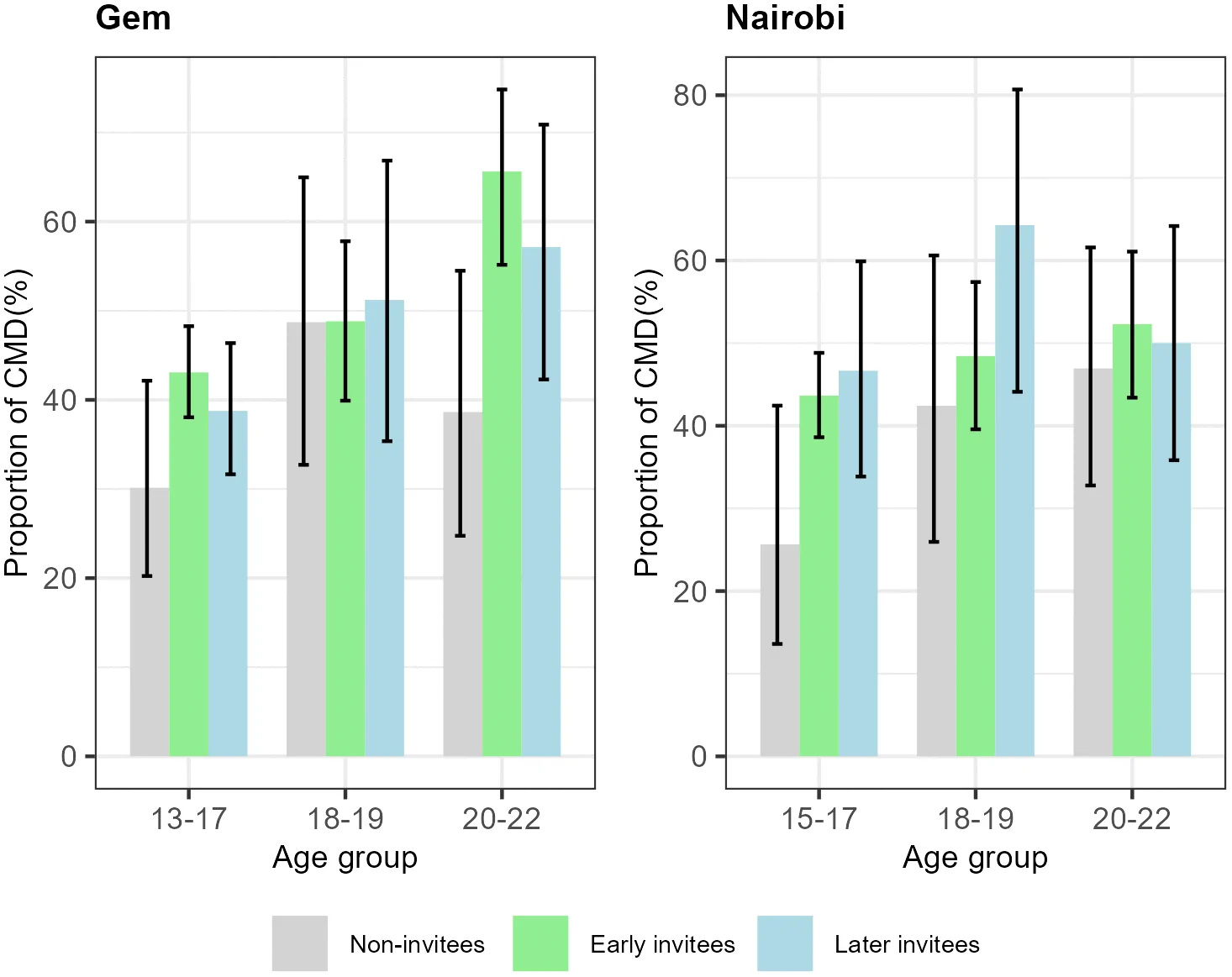
CMD prevalence in Gem and Nairobi in 2022, by age group at cohort enrolment and DREAMS invitation status.

Using a more stringent cut-off (≥10), 18% experienced CMD in Gem in 2022, while approximately 16% of AGYW experienced CMD in Nairobi. As with the cut-off of 7, CMD was more prevalent among older AGYW compared to younger AGYW in both settings. Among the older group, 22% in Gem and 20% in Nairobi experienced CMD in 2022, while among the younger age group 15% in Gem and 12% in Nairobi experienced CMD in 2022. CMD prevalence was higher among both early and later invitees when compared to non-invitees, in both Gem and Nairobi (Table B in S1 Appendix).

### CMD prevalence among AGYW, by calendar year

In both Gem and Nairobi, the overall prevalence of CMD was higher in 2022 than in 2019 and at the time of enrolment 4–5 years previously (in 2017/2018), mainly due to an increase in CMD prevalence among younger AGYW (Fig 4) and among early and later invitees (Fig 5). In Gem, the overall prevalence of CMD was 37% at enrolment in 2018 and 45% in 2022, while in Nairobi the prevalence was 41% at enrolment in 2017 and 46% in 2022 (Fig 4).

### Estimated impact of DREAMS on CMD prevalence

We assessed the experience of CMD among AGYW by comparing three different counterfactual scenarios; all were early invitees, all were later invitees, or all were non-invitees. In Gem, the estimated percentages of AGYW who experienced CMD were 38.4% if all were non-invitees, 48.9% if all were early invitees, and 45.1% if all were later invitees (Table 6). There was evidence of a difference between the percentages who experienced CMD when comparing early invitees with non-invitees (10.5%, 95% CI 1.5%, 20.8%). Differences among these three scenarios remained consistent when considering older and younger AGYW separately (and there was no evidence that the differences among the three scenarios differed by age group (p = 0.934)). Among younger AGYW, the estimated difference between early invitees and non-invitees was 9.7% (95% CI -3.3%,23.1%), while among older AGYW the estimated difference was 11.8% (95% CI -2.0%,24.5%) (Table 6).

**Table 6:** Impact of DREAMS on CMD prevalence in Gem and Nairobi.

	Estimated % if all AGYW are non-invitees (95% CI)	Estimated % if all AGYW are early invitees (95% CI)	Prevalence difference (95% CI)	Estimated % if all AGYW are later invitees (95% CI)	Prevalence difference (95% CI)
**Gem**					
Overall	38.4(29.8-46.8)	48.7(44.7-52.9)	**10.5(1.5 to 20.8)**	45.1(38.8-51.7)	**6.6(-3.8 to 16.9)**
13-17 years	34.2(22.0-45.9)	43.8(38.7-48.7)	**9.7(-3.2 to 23.1)**	39.0(31.3-47.1)	**4.8(-8.5 to 19.6)**
18-22 years	44.5(33.0-56.2)	56.3(49.6-62.6)	**11.8(-2.0 to 24.7)**	54.0(43.1-64.2)	**9.5(-5.8 to 24.5)**
**Nairobi**					
Overall	32.0(21.1-44.0)	46.4(42.4-50.6)	**14.4(2.1 to 26.4)**	55.3(43.1-68.7)	**23.3(5.8 to 41.2)**
15-17 years	15.9(5.5-30.2)	43.6(38.3-48.8)	**27.8(13.1to 39.2)**	45.7(28.1-63.1)	**29.9(9.2 to 50.6)**
18-22 years	47.4(31.4-62.6)	49.6(42.8-55.8)	**2.2(-14.7 to 19.4)**	64.3(50.7-77.9)	**16.8(-4.5 to 38.8)**

**Fig 4:**
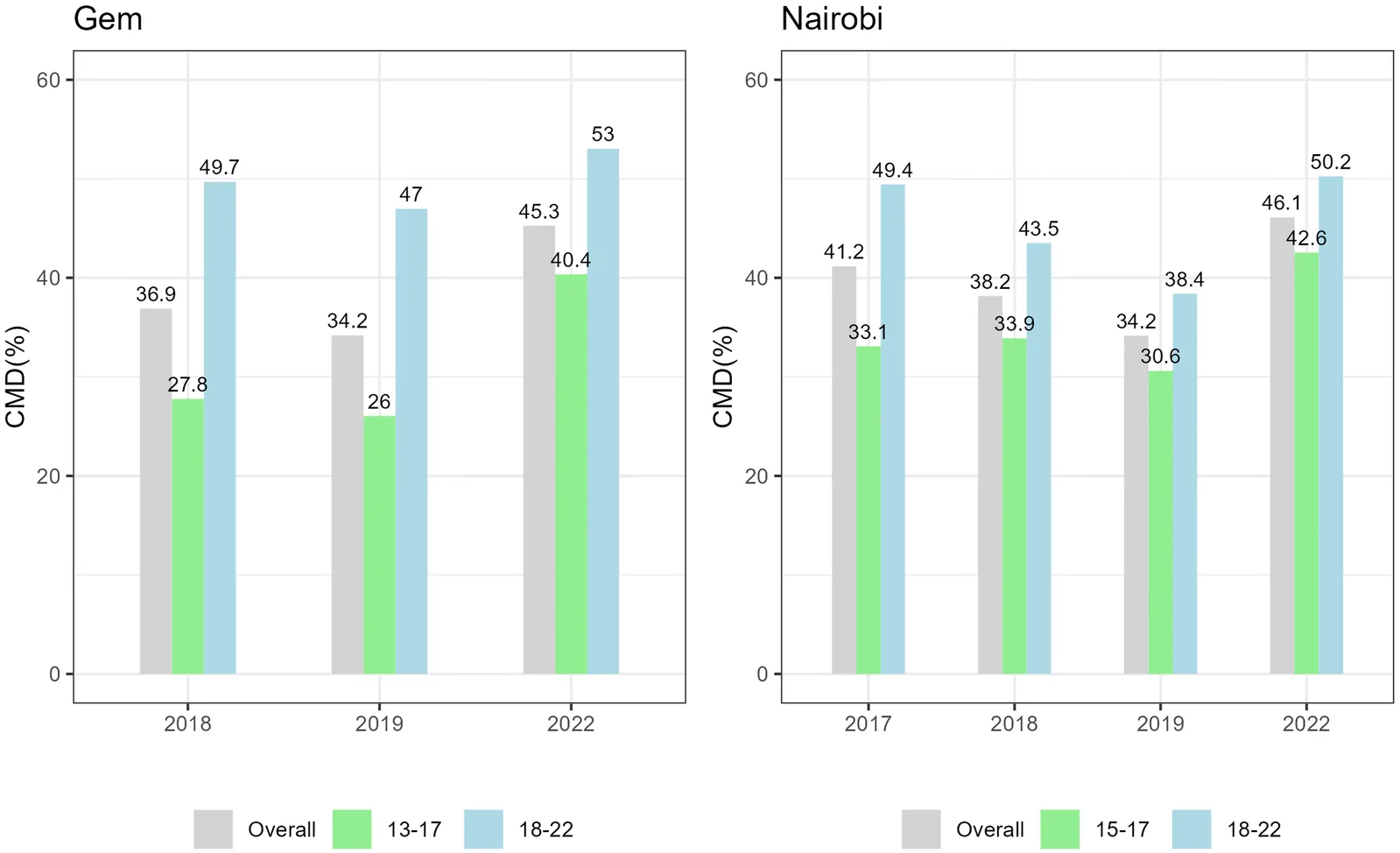
CMD prevalence in Gem and Nairobi, by interview year and age at cohort enrolment.

**Fig 5:**
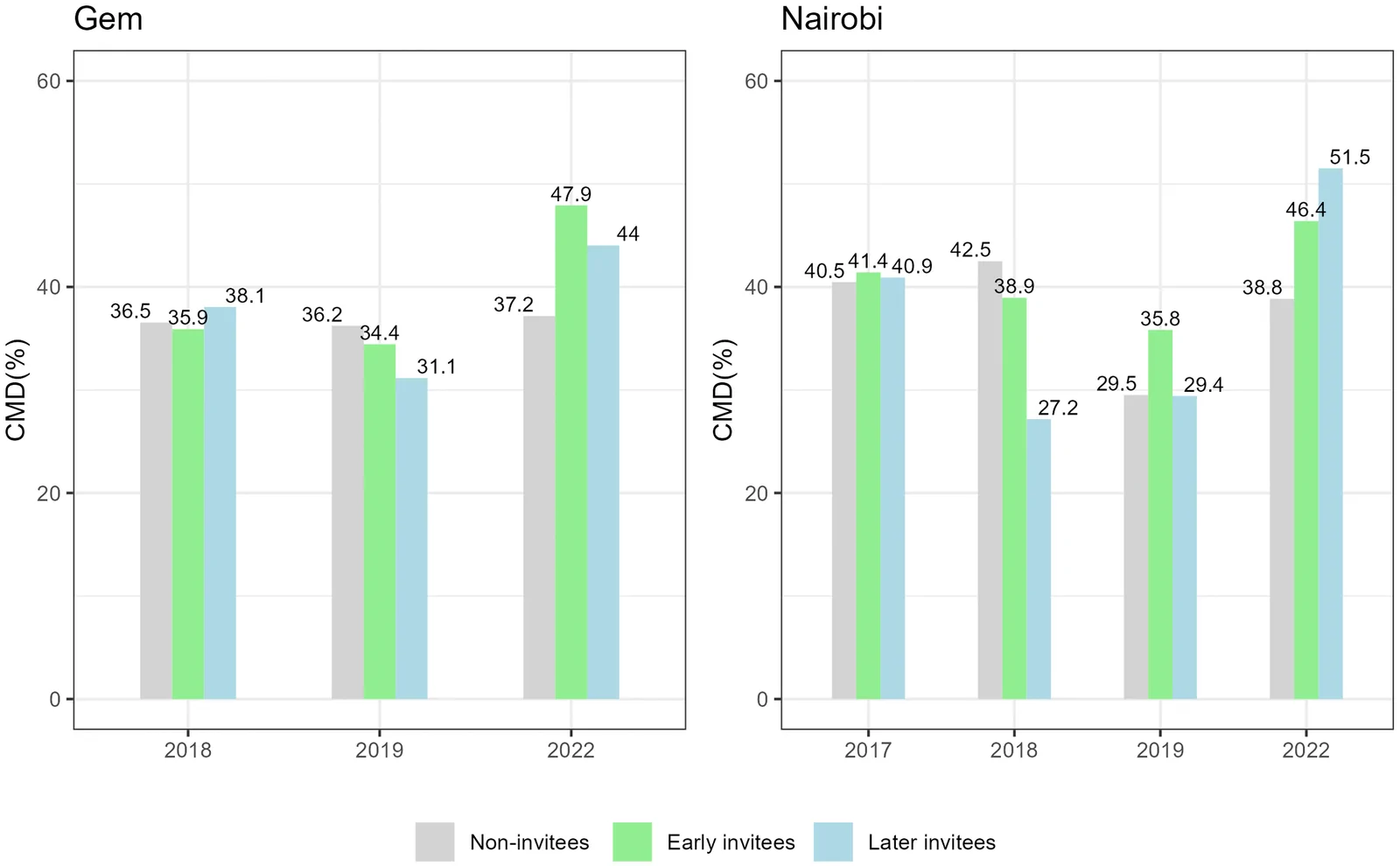
CMD prevalence in Gem and Nairobi, by interview year and invitation status.

In Nairobi, the estimated percentages of AGYW who experienced CMD were 32.0% if all were non-invitees, 46.4% if all were early invitees, and 55.3% if all were later invitees. There was evidence of a difference in experience of CMD when comparing early invitees to non-invitees (14.4, 95% CI 2.1%, 26.4%) and evidence of a difference in experience of CMD when comparing later invitees to non-invitees (23.3%, 95% CI 5.8%,41.2%). Similar to Gem, there was no evidence that the differences among invitees and non-invitees differed by age group (p = 0.414). Among the younger AGYW, there was evidence of a higher prevalence of CMD when comparing early invitees to non-invitees (27.8%, 95% CI 13.1%, 39.2%) and when comparing later invitees to non-invitees(29.9%, 95% CI 9.2%, 50.6%) (Table 6).The findings from these analyses were consistent with those from sensitivity analyses that used a more stringent cut-off to define CMD (Table 7).

**Table 7:** Impact of DREAMS on CMD prevalence in Gem and Nairobi. Sensitivity analysis (Stringent cut off ≥10).

	Estimated % if all AGYW are non-invitees (95% CI)	Estimated % if all AGYW are early invitees (95%CI)	Prevalence difference (95% CI)	Estimated % if all AGYW are later invitees (95% CI)	Prevalence difference (95% CI)
**Gem**					
Overall	38.1(29.3-46.1)	48.9(44.7-52.9)	**10.8(1.8 to 20.9)**	45.1(38.9-51.6)	**7.0(-3.2 to 17.3)**
13-17 years	33.6(21.1-44.4)	43.8(38.7-48.7)	**10.2(-2.0 to 24.3)**	39.0(31.1-46.8)	**5.4(-8.0 to 20.5)**
18-22 years	44.5(33.5-55.5)	56.3(49.6-62.6)	**11.8(-1.9 to 24.3)**	54.0(42.8-64.0)	**9.5(-6.7 to 24.2)**
**Nairobi**					
Overall	33.6(24.9-44.4)	46.4(42.5-50.7)	**12.8(1.8 to 22.1)**	55.6(45.8-63.8)	**22.0(6.8 to 34.6)**
15-17 years	18.4(7.5-34.2)	43.6(38.3-48.8)	**25.2(8.7 to 37.2)**	49.5(34.6-62.7)	**31.1(10.7 to 48.6)**
18-22 years	45.7(32.2-59.7)	49.7(42.9-55.8)	**4.0(-11.3 to 18.1)**	61.2(48.5-72.8)	**15.6(-5.6 to 33.6)**

The distribution of the IPTWs is shown in Supplementary Figure S2, Fig 2a and Fig 2b. A small number of weights exceeded the truncation threshold (n = 2, 0.02% of the sample in Gem and n = 25, 2.7% of the sample in Nairobi). The findings from the sensitivity analyses after truncation were very similar to the primary analysis findings (Table C in S1 Appendix).

## Discussion

### Key findings

With representative closed cohorts of AGYW followed between 2017/18 and 2022, in urban and rural settings of Kenya, we compared levels of CMD by exposure to the DREAMS program. In 2022, we found that a higher proportion of AGYW experienced CMD when we compared a scenario in which all AGYW were early invitees to DREAMS with a scenario in which all AGYW were non-invitees, in both settings. Specifically, the prevalence of CMD was about 10% higher in the scenario in which all AGYW were early invitees compared to the scenario in which all AGYW were non-invitees. In Nairobi, but less clearly in Gem, we also found that a higher proportion of AGYW experienced CMD when we compared a scenario in which all AGYW were later invitees with a scenario in which all were non-invitees. We also found a higher proportion of CMD when we compared scenarios in which all AGYW were early invitees versus non-invitees, and when all were later invitees versus non-invitees, in sub-group analyses among older and younger AGYW; these subgroup analyses should be interpreted with caution given the wide confidence intervals around the estimates.

CMD prevalence was high in both settings, at around 45%, and higher among the older AGYW (around 50%) compared to the younger AGYW (around 40%) in both settings. We also found that the prevalence of CMD was higher in 2022 than during 2017–2019, overall and specifically among DREAMS invitees and younger AGYW (aged 13–17 years at cohort enrolment in 2017/2018).

### Interpretation of findings

The finding that CMD prevalence was higher among DREAMS invitees than non-invitees was unexpected. First, social asset building was one of the 7 interventions comprising the primary package of DREAMS interventions in Kenya. This intervention was included to help AGYW to make connections with, and receive emotional and social support from, peers and female mentors and it aimed to create and foster a collective and connected identity among AGYW [21]. The intervention included mentor-led groupings of AGYW that met in safe spaces [21, 39, 50,51] and almost all DREAMS invitees participated in them [39, 50,51]. Our previous study (measuring outcomes in 2018 and 2019) indicated that DREAMS positively impacted social support in both Gem and Nairobi settings [25]. Also, analyses of qualitative data showed that many DREAMS participants highly valued their mentors, especially the efforts that mentors made to support them with individual problems. Second, parenting and caregiver interventions, classified within the secondary interventions category in the DREAMS package, aimed at promoting positive relationships between AGYW and parents or caregivers, with the ultimate goal of protecting AGYW against negative health and social outcomes [21]. Our previous study indicated that the uptake of parenting programs was relatively low in both settings, attributed to the challenges in the implementation and delivery of these interventions [52], but while their contribution may have been limited it was expected to be positive. Third, the uptake of school-based interventions, which were also included in the primary package of DREAMS interventions, was high among DREAMS invitees [52]. Although these interventions were focused on provision of accurate information on HIV prevention and building HIV prevention skills, empowering AGYW in this way might also empower them to take control of their overall health and this might extend to their mental health. Thus, a possible explanation for the higher CMD prevalence among DREAMS invitees compared with non-invitees is that they had greater awareness or recognition of their own mental health, and greater willingness to disclose symptoms of mental ill-health, compared with non-invitees. DREAMS may have created an environment that helped AGYW to recognize and articulate their mental health challenges better, for example through mentorship, safe spaces, life skills training and fostering more open relationships with parents or guardians. These challenges might otherwise have been concealed due to shame or fear.

On the other hand, there is a possibility that DREAMS contributed to the experience of CMD among some AGYW. In the 2022 cohort interviews, around 60% of DREAMS invitees in Gem and around 30% of DREAMS invitees in Nairobi reported that a negative experience of DREAMS was unfairness, with some invitees getting benefits while others did not; the sense of unfairness was particularly in terms of secondary interventions that provided valuable material resources (for example, financial support towards school fees). Additionally, some AGYW experienced negative reactions from partners or husbands due to their participation in DREAMS or changes in their behaviour, such as increased independence. Furthermore, some DREAMS invitees may have felt judged by adolescent boys and young men, who were vocal about not being included in DREAMS interventions. However, there is no evidence to suggest that these experiences directly caused CMD.

It is also possible that there is residual confounding of our comparisons between invitees and non-invitees, especially in the context that by 2022 around 85% of AGYW in the cohort had been invited to participate in DREAMS and only 15% remained as non-invitees. The non-invitees may be different to the invitees in terms of characteristics that affect mental health but which we have not controlled for in our analyses.

Between the 2019 and 2022 evaluations, CMD prevalence increased. This could be attributed to several factors, including the impact of the COVID-19 pandemic, whose social and economic stresses may have exacerbated mental health issues, and the fact that in 2022 the cohort was several years older than in 2019. A study in Kenya showed that approximately 30% of young women reported symptoms consistent with probable depression during the pandemic, while qualitative evidence linked these high rates directly to pandemic-related isolation, economic hardship, and disruptions to daily life [53]. Additionally, findings from a qualitative study also showed that the challenges posed by COVID-19 such as school closures, exposure to violence, and socio-economic stressors contributed to a rise in mental health issues, especially among adolescent girls [54]. These pandemic-related stressors may have contributed to the rise in CMD over time, independent of DREAMS exposure.

### Findings in context

Impacts of DREAMS specifically on CMD have not been reported elsewhere. Several previous studies have suggested a positive impact of individual, community, and family-based interventions, and digitally-based interventions, on CMD, and have suggested that exposure to these interventions reduced CMD [32,55]. A systematic review on the effect of a series of school-based interventions on CMD found no evidence of an impact of these interventions [56], while another systematic review on the impact of school-based preventive interventions also found no evidence of an impact on CMD symptoms such as stress and the lack of impact in this study was attributed to low uptake of these services [57]. A study in Uganda that evaluated the effect of family economic empowerment and multiple family group interventions on CMD and compared the interventions found no evidence of a difference between the two interventions; this was attributed to a scarcity of resources [58]. Results from a randomized controlled trial in Kenya that evaluated the impact of a church-based intervention for families found no effect on CMD among adolescents [59].

Our findings complement this body of research evaluating the impact of multi-component interventions on CMD, extending them to findings from two Kenyan settings about the impact of a multi-component intervention (DREAMS) that focussed on improving health and social outcomes among AGYW. Kenya encounters legislative and financial obstacles that hinder the effective implementation of its mental health policies, resulting in a persistent gap in mental health care and treatment [34]. Thus, the high prevalence of CMD in our study settings also reflects insufficient mental health services and support systems in the region, and the inaccessibility of these services. The high prevalence of CMD in contexts of urban informal settlement areas in Nairobi and rural Gem in western Kenya, as observed in other settings in SSA [5,60], also highlights the role of social determinants of health (such as poverty, social inequality, and lack of access to education and employment opportunities) in contributing to mental health disparities between places.

The high prevalence of CMD, particularly among the older AGYW, reflects the specific challenges faced by this age group. These challenges include multiple and sometimes conflicting demands such as caring for children or engaging in income-generating activities, which may contribute to anxiety and depression. DREAMS provided a comprehensive package to address multiple vulnerabilities among AGYW, but there was no intervention specifically on mental health. Therefore, the high prevalence of CMD in the two settings indicates that early interventions and mental health promotion initiatives would be valuable and that CMD-specific interventions should be incorporated within health and HIV prevention programmes such as DREAMS.

While some of the observed effects may appear modest, they could be of practical importance. For instance, even small reductions in CMD prevalence can have significant short-term benefits, such as improved functioning, and better engagement in school or health programmes. In the longer term, lowering CMD prevalence can improve life-course opportunities, improve health outcomes and lessen the overall burden on health systems.

### Study strengths and limitations

Representative samples of AGYW were drawn from established health demographic surveillance platforms, facilitating the generalisability of findings, and high retention in the cohort study limited selection bias. We used inverse probability of treatment weighting (IPTW) based on propensity scores to estimate the causal effect of DREAMS interventions on CMD while adjusting for time-dependent confounding and there were no missing data in the key confounding variables used in this analysis. We used stabilised weights and examined their distribution, and we found no evidence of extreme values. Findings in the sensitivity analyses were broadly consistent with the findings from the primary analysis, indicating the robustness of our results to different thresholds used to define CMD and to limiting (or not) the weight an individual AGYW was given in the causal analysis. We also used a validated tool in the measurement of CMD. Although this tool does not provide a clinical diagnosis of CMD, various studies have validated the criteria used to determine the cut-off (for defining CMD, yes or no) in settings that are similar to those of the current study [41,42], and a sensitivity analysis using a more stringent cut-off gave similar conclusions. CMD symptoms were self-reported, and there were changes in mode of data collection and place of interview administration in 2022 compared with 2017–2019, but the research assistants who collected the data were well trained and this will have limited the possibility of misunderstandings or misreporting.

Limitations include the potential for residual confounding in our comparisons between early and later invitees and non-invitees due to unmeasured factors at the time of cohort enrolment and follow-up. For example, marital status was not measured in Gem at cohort enrolment, and it might be associated with whether or not an AGYW was invited to DREAMS and also associated with CMD. If unmeasured factors are associated with both invitation to DREAMS and CMD and were not accounted for, our effect estimates may be over or underestimated. However, we attempted to measure and control for known individual and household characteristics that were used by implementing partners to guide who was invited to DREAMS. Although we adjusted for a comprehensive set of characteristics, these variables may not fully capture the context-specific criteria used in the Girl Roster method for invitation to DREAMS. As a result, residual confounding due to unmeasured factors cannot be ruled out. Additionally, we did not adjust for CMD measured at cohort enrolment because it was not an eligibility criterion for invitation to DREAMS and could not be considered a confounder. Furthermore, for some early invitees, cohort enrolment happened after invitation to DREAMS and as a result, CMD at enrolment does not consistently represent a pre-exposure confounder.

By 2022, 85% of AGYW in the cohort had been invited to DREAMS and only 15% remained as non-invitees, so our comparison group of non-invitees was relatively small as well as potentially being different to invitees for characteristics that are determinants of mental health but which we did not control for in our analyses; our comparisons would have been more robust if the comparison group of non-invitees had been larger. There was also differential loss to follow-up by AGYW characteristics at cohort enrolment, potentially contributing to selection bias, but the high retention in the cohort will have limited the extent of this bias. Nonetheless, it remains plausible that differences in outcomes existed between individuals who were followed up and those who were not, even after accounting for their characteristics at enrolment. For example, if AGYW with poorer mental health outcomes were less likely to be followed up, our findings might underestimate the true prevalence of CMD and the potential effect of the interventions. Although we used inverse probability of censoring weighting to account for differential loss to follow-up, and we adjusted for a set of characteristics associated with follow-up, unmeasured predictors of loss to follow-up may remain and could result in residual selection bias.

There is also a possibility of measurement error of both outcome (CMD) and invitation to DREAMS due to our reliance on self-reported data, which may be subject to recall bias and misclassification. For instance, the proportion categorized as DREAMS invitees may be underestimated if some AGYW did not self-identify as being invited to participate in DREAMS activities. However, the exposure misclassification is expected to be small in both study settings since the invitation to DREAMS activities was a formalised process that was coordinated by a single implementing partner, and the eligibility criteria were clear and well-defined. The invitation process was therefore well understood by both implementers and invitees, making misclassification unlikely. Similarly, the symptoms of CMD were based on self-reported screening tools rather than clinical diagnoses, which may lead to under or overestimation of true CMD prevalence. CMD was measured using the self-reported Shona Symptom Questionnaire and may therefore be subject to outcome misclassification due to reporting bias or differences in participants’ interpretation of symptoms. If such misclassification was non-differential with respect to DREAMS invitation, it would be expected to attenuate the estimated associations towards the null. However, the Shona Symptom Questionnaire has been widely validated in similar settings, and the variation in CMD prevalence observed across participant characteristics in our study suggests that the instrument was able to differentiate between groups with differing levels of CMD.

A further consideration is the timing of the outcome assessment. CMD was measured in 2022, which for later invitees was relatively soon after invitation and so reflects a more immediate outcome. While we found no evidence of a reduction in CMD among later invitees, for early invitees we cannot rule out the possibility that there were short-term effects that had attenuated by the time of the outcome assessment in 2022. However, DREAMS was designed to address multiple structural, social, and behavioural determinants of health, and it was intended to have lasting effects on the AGYW who participated in the programme.

Additionally, the potential for reverse causation is minimal. Our study was explicitly designed to estimate the impact of DREAMS invitation on CMD outcomes, with a clearly defined temporal relationship between exposure and outcome. Importantly, AGYW were invited to participate in DREAMS based on socio-economic and sexual risk characteristics, and not on the basis of their mental health status.

It is possible that there were some spillover effects from invitees to AGYW who were non-invitees, for example through peer influence, community-wide components of DREAMS, or shared services in households or schools. Such spillover effects could attenuate observed differences between invitees and non-invitees potentially biasing the estimated effects towards the null. However, we believe the spillover effects will have been limited for the outcome of CMD, because this outcome is largely influenced by individual experiences and structural vulnerabilities.

There is the potential for programmatic contamination, when evaluating the effect of a particular intervention, as other interventions that targeted AGYW could have been delivered in the study settings. AGYW who were not invited to DREAMS could have accessed services through alternative programs, potentially reducing the observed effect of DREAMS. However, this effect (of programmatic contamination) is likely minimal in this study, as DREAMS was the primary multi-sectoral intervention been delivered to AGYW during the study period and it was distinct from other programs.

While it is possible that certain confounding variables measured at cohort enrolment may have already been influenced by DREAMS (which started in 2016), it is unlikely that such change was substantial given the time it took for DREAMS implementation to be fully established. Nonetheless, if such influence occurred, it could lead to an underestimation of the true effect of DREAMS invitation on CMD.

There is a possibility that the COVID-19 pandemic may also have contributed to residual confounding. We expect any resulting bias to be limited because we adjusted for a range of socio-economic and behavioural characteristics associated with both DREAMS invitation and CMD, which are also likely to have influenced individuals’ experiences of the pandemic In addition, although the extent to which individuals were affected by the pandemic may have varied, the pandemic affected the study population broadly and was therefore unlikely to have resulted in substantial differential confounding. Furthermore, invitation to DREAMS was largely determined before or independently of the pandemic, reducing the likelihood that differential pandemic experiences substantially influenced the estimated effects.

Finally, our findings should be interpreted considering random error. As with any study based on a sample rather than the entire population, the estimated effects are subject to sampling variability. Although we present 95% confidence intervals to quantify this uncertainty, the observed estimates remain compatible with a range of plausible effect sizes under the assumptions of the analysis.

Although our study was conducted in two Kenyan settings, the findings are likely to be broadly generalisable to similar DREAMS implementation contexts in East Africa. Our results may not be generalisable to all countries where DREAMS is implemented, but because they represent diverse implementation contexts, they can still contribute important insights for other settings in which DREAMS (or other combination HIV prevention) interventions are being delivered.

## Conclusions

We found that the prevalence of CMD was higher among DREAMS invitees than non-invitees, for both early and later invitees and in both Gem and Nairobi. A plausible explanation for this finding is that DREAMS increased AGYW’s awareness and recognition of, and willingness to disclose and report, CMD symptoms. Our findings emphasize the importance of strengthening DREAMS by incorporating CMD-specific interventions. Such interventions were not a core component of DREAMS, but they have been introduced in a few intervention sites in Kenya since 2023. Building on this progress, we recommend integrating mental health services (for example, cognitive behavioral therapy (CBT)-based group sessions) and peer support into the DREAMS package, delivered through existing safe spaces. DREAMS mentors should be trained in mental health literacy, identification of poor mental health indicators, and appropriate referral for mental health support. While this will incur additional costs, these could be modest and might result in substantial benefits, including improved mental well-being and improved HIV prevention outcomes. Moreover, it is feasible to integrate some mental health services through gradual adaptation and utilisation of existing infrastructure, like schools and safe spaces.

## Supporting information

S1 Appendix Supplementary material. Table A: Mental Health questions. Table B: CMD prevalence in Gem and Nairobi in 2022, overall and by age group at cohort enrolment and DREAMS invitation status. Figure A: Propensity score distribution in Gem. Figure B: Propensity score distribution in Nairobi. Figure C: Distribution of the stabilised inverse treatment probability weights (IPW), 2022 in Gem. Figure D: Distribution of the stabilised inverse treatment probability weights (IPW), 2022 in Nairobi. Figure E: Covariate balance plot: Compares non-invitees and early invitees in Gem. Figure F: Covariate balance plot: Compares non-invitees and later invitees in Gem. Figure G: Covariate balance plot: Compares non-invitees and early invitees in Nairobi. Figure H: Covariate balance plot: Compares non-invitees and later invitees in Nairobi. Table C: Impact of DREAMS on CMD prevalence in Gem and Nairobi. Sensitivity analysis (IPTW truncation). Table D: Predictors of retention into the cohort in 2022 using characteristics of AGYW as at 2017 in Nairobi. Table E: Predictors of retention into the cohort in 2022 using characteristics of AGYW as at 2018 in Gem
(DOCX)

S1 Checklist Inclusivity in global research. (DOCX)
