# Supplementary material for: Impact of the DREAMS interventions on Common mental disorders among adolescent girls and young women: Causal analysis of a prospective cohort in two Kenyan settings
Source: PLOS Ment Health. 2026 Nov 3;3(11):e0000765. doi: 10.1371/journal.pmen.0000765 (PMC13730345; doi:10.1371/journal.pmen.0000765)
Supplement: Supplementary file 1 [file pmen.0000765.s001.docx]

**Table A: Mental Health questions**

a. Did you have times in which you were thinking deeply or thinking about many things?
b. Did you find yourself sometimes failing to concentrate?
c. Did you lose your temper or get annoyed over trivial matters?
d. Did you have nightmares of bad dreams?
e. Did you sometimes see or hear things that others could not see or hear?
f. Did trivial things frighten you?
g. Did you sometimes fail to sleep or lose sleep?
h. Were there moments when you felt life was so tough that you cried or wanted to cry?
i. Did you feel run down (tired)?
j. Did you at times feel like committing suicide?
k. Were you generally unhappy with things you were doing each day?
l. Was your work lagging behind?
m. Did you feel you had problems in deciding what to do?
n. Was your stomach aching? **excluded from the questionnaire**

**Source: (Patel, Simunyu et al. 1997)(58) (Responses; Yes/No, scope; in the past week)*

| **Table B: CMD prevalence in Gem and Nairobi in 2022, overall and by age group at cohort enrolment and DREAMS invitation status** | | | | | | | | |  | |
| --- | --- | --- | --- | --- | --- | --- | --- | --- | --- | --- |
|  | **Overall** | | **Non-invitees** | | **Early invitees** | | **Later invitees** | | |  |
|  | **n/N** | **%** | **n/N** | **%** | **n/N** | **%** | **n/N** | **%** | |  |
| **Gem** |  |  |  |  |  |  |  |  | |  |
| **CMD** (SSQ ≥ 10) |  |  |  |  |  |  |  |  | |  |
| Overall (total) | 179/1023 | **17.5** | 15/156 | **9.6** | 116/599 | **19.4** | 48/268 | **17.9** | |  |
| 13-17 years | 92/627 | **14.7** | 5/73 | **6.8** | 62/376 | **16.5** | 25/178 | **14.0** | |  |
| 18-22 years | 87/396 | **22.0** | 10/83 | **12.0** | 54/223 | **24.2** | 23/90 | **25.6** | |  |
| **Nairobi** |  |  |  |  |  |  |  |  | |  |
| **CMD** (SSQ ≥10) |  |  |  |  |  |  |  |  | |  |
| Overall (total) | 143/889 | **16.1** | 17/121 | **14.0** | 103/636 | **16.2** | 23/132 | **17.4** | |  |
| 15-17 years | 59/477 | **12.4** | 2/39 | **5.1** | 48/378 | **12.7** | 9/60 | **15.0** | |  |
| 18-22 years | 84/412 | **20.4** | 15/82 | **18.3** | 55/258 | **21.3** | 14/72 | **19.4** | |  |

**Figure A: Propensity score distribution in Gem**

**Figure B: Propensity score distribution in Nairobi**

**Figure C: Distribution of the stabilised inverse treatment probability weights(IPW), 2022 in Gem**

**Figure D: Distribution of the stabilised inverse treatment probability weights(IPW), 2022 in Nairobi**

**Figure E: Covariate balance plot: Compares non-invitees and early invitees in Gem**

**Figure F: Covariate balance plot: Compares non-invitees and later invitees in Gem**

**Figure G: Covariate balance plot: Compares non-invitees and early invitees in Nairobi**

**Figure H: Covariate balance plot: Compares non-invitees and later invitees in Nairobi**

**Table C**: **Impact of DREAMS on CMD prevalence in Gem and Nairobi.** **Sensitivity analysis (IPTW truncation)**

|  | **Estimated % if all AGYW are non-invitees(95% CI)** | **Estimated % if all AGYW are early invitees(95% CI)** | **Prevalence difference(95% CI)** | **Estimated % if all AGYW are later invitees(95% CI)** | **Prevalence difference(95% CI)** |
| --- | --- | --- | --- | --- | --- |
| **Gem** |  |  |  |  |  |
| Overall | 11.3(5.6-17.5) | 19.8(16.6-23.0) | **8.5(2.1 to 15.4)** | 19.6(14.3-24.5) | **8.2(-0. 2 to 16.1)** |
| 13-17 years | 10.5(1.8-20.1) | 16.6(12.9-20.1) | **6.1(-4.1 to 15.5)** | 15.5(9.9-21.9) | **5.0(-6.7 to 15.8)** |
| 18-22 years | 12.5(5.6-20.6) | 24.5(18.8-30.4) | **12.0(1.4 to 21.2)** | 25.5(16.4-34.3) | **13.0(0.4 to 23.4)** |
| **Nairobi** |  |  |  |  |  |
| Overall | 9.8(4.9-16.1) | 16.8(13.8-19.8) | **6.9(-0.2 to 12.8)** | 18.7(8.9-35.0) | **8.9(-3.5 to 25.8)** |
| 15-17 years | 2.2(0.0-7.2) | 12.3(9.2-15.6) | **10.1(4.2 to 14.7)** | 15.4(5.7-27.9) | **13.2(2.7 to 26.8)** |
| 18-22 years | 17.0(8.4-28.0) | 21.5(16.6-27.3) | **4.5(-7.3 to 14.8)** | 21.7(7.0-45.3) | **4.7(-15.0 to 30.0)** |

| **Table D: Predictors of retention in to the cohort in 2022 using characteristics of AGYW as at 2017 in Nairobi** | | | | | | | |
| --- | --- | --- | --- | --- | --- | --- | --- |
|  | *n/N* | *Row %* | *unadjusted OR(95%CI)* | *p value* | *adjusted OR (95% CI)* | *p value* | *aOR model (95% CI)* |
|  |  |  |  |  |  |  |  |
| **DSS study site** |  |  |  |  |  |  |  |
| Korogocho | 515/617 | 83,5 | 1 |  | 1 |  | 1 |
| Viwandani | 374/464 | 80,6 | 0.82(0.60-1.13) | 0,224 | 0.99 (0.63-1.57) | 0,978 | 0.88(0.64-1.22) |
| **Age group cohort: 2017** |  |  |  |  |  |  |  |
| 15-17 | 477/547 | 87,2 | 1 |  | 1 |  | 1 |
| 18-22 | 412/534 | 77,2 | 0.50(0.36-0.68) | <0.001 | 0.97 (0.58-1.62) | 0,911 | 0.86(0.57-1.29) |
| **Invited to participate in DREAMS** |  |  |  |  |  |  |  |
| not invited | 420/545 | 77,1 | 1 |  | 1 |  | 1 |
| invited | 469/536 | 87,5 | 2.08(1.51-2.88) | <0.001 | 1.45 (1.00-2.11) | 0,05 | 2.05(1.47-2.85) |
| **Currently in school: 2017** |  |  |  |  |  |  |  |
| No | 338/455 | 74,3 | 1 |  | 1 |  |  |
| Yes | 551/626 | 88 | 2.54(1.85-3.50) | <0.001 | 1.37 (0.82-2.27) | 0,231 |  |
| **Highest education level completed** |  |  |  |  |  |  |  |
| None/incomplete primary | 105/124 | 84,7 | 1 |  | 1 |  |  |
| Complete primary | 167/217 | 77 | 0.60(0.34-1.08) | 0,074 | 0.66 (0.34-1.28) | 0,223 |  |
| Some secondary | 416/491 | 84,7 | 1(0.58-1.73) |  | 0.68 (0.36-1.28) |  |  |
| Complete secondary/Tertiary | 201/249 | 80,7 | 0.76(0.42-1.36) |  | 0.89 (0.44-1.81) |  |  |
| **Marital status (2 groups) 2017** |  |  |  |  |  |  |  |
| never married | 728/843 | 86,4 | 1 |  | 1 |  |  |
| prev(curr) married/living w partner | 161/238 | 67,6 | 0.33(0.24-0.46) | <0.001 | 0.43 (0.22-0.82) | 0,011 |  |
| **Re-grouped religion of AGYW: 2017** |  |  |  |  |  |  |  |
| No religion/other | 20/23 | 87 | 1 |  | 1 |  |  |
| Catholic | 255/309 | 82,5 | 0.71(0.20-2.47) | 0,028 | 0.76 (0.19-2.95) | 0,689 |  |
| Other Christian | 487/608 | 80,1 | 0.60(0.18-2.06) |  | 0.68 (0.18-2.59) |  |  |
| Muslim | 127/141 | 90,1 | 1.36(0.36-5.16) |  | 1.23 (0.29-5.31) |  |  |
| **Food insecurity** |  |  |  |  |  |  |  |
| No | 593/730 | 81,2 | 1 |  | 1 |  |  |
| Yes | 296/351 | 84,3 | 1.24(0.88-1.75) | 0,208 | 1.18 (0.78-1.80) | 0,435 |  |
| **Wealth quantile** |  |  |  |  |  |  |  |
| Poor | 299/361 | 82,8 | 1 |  | 1 |  |  |
| Medium | 297/360 | 82,5 | 0.98(0.66-1.44) | 0,87 | 1.15 (0.72-1.83) | 0,552 |  |
| Wealthy | 293/360 | 81,4 | 0.91(0.62-1.33) |  | 1.24 (0.72-2.13) |  |  |
| **Self-assessed household poverty, 2017** |  |  |  |  |  |  |  |
| Very poor | 115/138 | 83,3 | 1 |  | 1 |  |  |
| Moderately poor | 703/859 | 81,8 | 0.90(0.56-1.46) | 0,771 | 0.98 (0.57-1.67) | 0,933 |  |
| Not poor | 71/84 | 84,5 | 1.09(0.52-2.29) |  | 1.09 (0.47-2.50) |  |  |
| **Orphanhood status** |  |  |  |  |  |  |  |
| Not an orphan | 656/744 | 88,2 | 1 |  | 1 |  |  |
| Single/double orphan | 170/209 | 81,3 | 0.58(0.39-0.88) | <0.001 | 0.64 (0.42-0.99) | 0,044 |  |
| Unknown/missing | 63/128 | 49,2 | 0.13(0.09-0.20) |  | 0.17 (0.11-0.27) |  |  |
| **Lived elsewhere for >1mo in last 3 mo** |  |  |  |  |  |  |  |
| No | 732/894 | 81,9 | 1 |  | 1 |  |  |
| Yes | 157/187 | 84 | 1.16(0.76-1.77) | 0,494 | 1.01 (0.62-1.64) | 0,962 |  |
| **Access to credit: 2017** |  |  |  |  |  |  |  |
| No | 449/550 | 81,6 | 1 |  | 1 |  |  |
| Yes | 395/482 | 82 | 1.02(0.74-1.40) | 0,144 | 0.94 (0.66-1.34) | 0,731 |  |
| Don't know | 45/49 | 91,8 | 2.53(0.89-7.20) |  | 1.76 (0.57-5.42) |  |  |
| **Ever had sex, ever pregnant composite var, 2017** |  |  |  |  |  |  |  |
| Never had sex | 564/644 | 87,6 | 1 |  | 1 |  | 1 |
| Ever sex, never preg | 108/138 | 78,3 | 0.51(0.32-0.81) | <0.001 | 0.89 (0.49-1.63) | 0,711 | 0.63(0.38-1.05) |
| Ever pregnant | 217/299 | 72,6 | 0.38(0.27-0.53) |  | 1.36 (0.65-2.87) |  | 0.48(0.31-0.74) |
| **Highest educational level of the HHH: 2017** |  |  |  |  |  |  |  |
| None/incomplete primary | 202/237 | 85,2 | 1 |  | 1 |  |  |
| Complete primary | 217/267 | 81,3 | 0.75(0.47-1.21) | 0,111 | 1.01 (0.59-1.74) | 0,967 |  |
| Incomplete primary | 76/100 | 76 | 0.55(0.31-0.98) |  | 1.02 (0.51-2.03) |  |  |
| Complete primary | 220/274 | 80,3 | 0.71(0.44-1.13) |  | 1.19 (0.68-2.09) |  |  |
| Complete tertiary | 58/72 | 80,6 | 0.72(0.36-1.42) |  | 1.15 (0.52-2.57) |  |  |
| Don't know | 116/131 | 88,5 | 1.34(0.70-2.56) |  | 1.46 (0.72-2.94) |  |  |
| **Experience of violence** |  |  |  |  |  |  |  |
| No | 500/607 | 82,4 | 1 |  | 1 |  |  |
| Yes | 389/474 | 82,1 | 0.98(0.72-1.34) | 0,897 | 1.29 (0.90-1.86) | 0,172 |  |
| **Experienced any household shocks in the last 12 months** |  |  |  |  |  |  |  |
| No | 303/374 | 81 | 1 |  | 1 |  |  |
| Yes | 586/707 | 82,9 | 1.13(0.82-1.57) | 0,446 | 1 (0.68-1.46) | 0,99 |  |
| **Generalised self efficaccy:2017** |  |  |  |  |  |  |  |
| Low self efficacy | 506/617 | 82 | 1 |  | 1 |  |  |
| High self efficacy | 383/464 | 82,5 | 1.04(0.76-1.42) | 0,820 | 0.96 (0.67-1.37) | 0,816 |  |
|  |  |  |  |  |  |  |  |

| **Table E: Predictors of retention in to the cohort in 2022 using characteristics of AGYW as at 2018 in Gem** | | | | | | | |
| --- | --- | --- | --- | --- | --- | --- | --- |
|  | *n/N* | *Row %* | *unadjusted OR(95%CI)* | *p value* | *adjusted OR (95% CI)* | *p value* | *aOR model (95% CI)* |
|  |  |  |  |  |  |  |  |
| **Invitation to participate in DREAMS, 2018** |  |  |  |  |  |  |  |
| No | 427/514 | 83,1 | 1 |  | 1 |  | 1 |
| Yes | 600/657 | 91,3 | 2.14(1.50-3.06) | <0.001 | 1.85 (1.26-2.71) | 0,002 | 1.96(1.36-2.81) |
| **Age group, 2018** |  |  |  |  |  |  |  |
| 13-17 | 628/684 | 91,8 | 1 |  | 1 |  | 1 |
| 18-22 | 399/487 | 81,9 | 0.40(0.28-0.58) | <0.001 | 0.68 (0.41-1.11) | 0,125 | 0.69(0.44-1.07) |
| **Education level, 2018** |  |  |  |  |  |  |  |
| None/Primary | 440/481 | 91,5 | 1 |  | 1 |  |  |
| Secondary and above | 359/411 | 87,3 | 0.64(0.42-0.99) | 0,046 | 0.85 (0.51-1.41) | 0,531 |  |
| Unknown | 228/279 | 81,7 | 0.42(0.27-0.65) | <0.001 | 0.78 (0.47-1.31) |  |  |
| **SES and food insecurity composite variable** |  |  |  |  |  |  |  |
| Food insecure, low medium high SES | 227/253 | 89,7 | 1 |  | 1 |  |  |
| Food secure, low SES | 225/262 | 85,9 | 0.70(0.41-1.19) | 0,185 | 0.87 (0.49-1.53) | 0,621 |  |
| Food secure, medium SES | 189/210 | 90 | 1.03(0.56-1.89) | 0,922 | 1.16 (0.61-2.22) |  |  |
| Food secure, high SES | 386/446 | 86,5 | 0.74(0.45-1.20) | 0,221 | 0.82 (0.47-1.43) |  |  |
| **Self-assessed poverty of household, 2018** |  |  |  |  |  |  |  |
| Very Poor | 137/151 | 90,7 | 1 |  | 1 |  |  |
| Moderately poor | 734/844 | 87 | 0.68(0.38-1.22) | 0,2 | 0.92 (0.49-1.74) | 0,794 |  |
| Not Poor | 156/176 | 88,6 | 0.80(0.39-1.64) | 0,537 | 1.08 (0.48-2.43) |  |  |
| **Orphanhood** |  |  |  |  |  |  |  |
| No | 623/687 | 90,7 | 1 |  | 1 |  |  |
| Maternal | 36/40 | 90 | 0.92(0.32-2.68) | 0,885 | 0.97 (0.32-2.95) | 0,958 |  |
| Paternal | 96/107 | 89,7 | 0.90(0.46-1.76) | 0,751 | 0.94 (0.46-1.90) |  |  |
| Total | 32/39 | 82,1 | 0.47(0.20-1.11) | 0,084 | 0.75 (0.30-1.86) |  |  |
| Unknown | 240/298 | 80,5 | 0.43(0.29-0.62) | <0.001 | 0.49 (0.31-0.77) |  |  |
| **Ever had sex, ever pregnant composite variable, 2018** |  |  |  |  |  |  |  |
| Never had sex | 705/766 | 92 | 1 |  | 1 |  | 1 |
| Ever sex,never pregnant | 161/190 | 84,7 | 0.48(0.30-0.77) | 0,002 | 0.58 (0.34-0.99) | 0,047 | 0.60(0.36-1.00) |
| Ever pregnant | 161/215 | 74,9 | 0.26(0.17-0.39) | <0.001 | 0.34 (0.20-0.57) |  | 0.36(0.22-0.58) |
| **Access to credit, 2018** |  |  |  |  |  |  |  |
| No | 517/580 | 89,1 | 1 |  | 1 |  |  |
| Yes | 320/367 | 87,2 | 0.83(0.55-1.24) | 0,363 | 0.76 (0.50-1.17) | 0,218 |  |
| Don't know' | 190/224 | 84,8 | 0.68(0.43-1.07) | 0,093 | 0.45 (0.27-0.75) |  |  |
| **Highest level of education of head of household** |  |  |  |  |  |  |  |
| No schooling/incomplete primary | 212/245 | 86,5 | 1 |  | 1 |  |  |
| Complete primary | 238/263 | 90,5 | 1.48(0.85-2.57) | 0,162 | 1.69 (0.94-3.01) | 0,077 |  |
| Incomplete/complete secondary | 225/269 | 83,6 | 0.80(0.49-1.30) | 0,36 | 0.97 (0.57-1.67) |  |  |
| Tertiary | 88/101 | 87,1 | 1.05(0.53-2.10) | 0,882 | 1.11 (0.51-2.38) |  |  |
| Don't know | 264/293 | 90,1 | 1.42(0.83-2.41) | 0,198 | 1.35 (0.77-2.39) |  |  |
| **Experience of Household shocks** |  |  |  |  |  |  |  |
| No | 686/783 | 87,6 | 1 |  | 1 |  |  |
| Yes | 341/388 | 87,9 | 1.03(0.71-1.49) | 0,893 | 1.21 (0.81-1.81) | 0,346 |  |
| **Experienced violence, 2018** |  |  |  |  |  |  |  |
| No | 847/961 | 88,1 | 1 |  | 1 |  |  |
| Yes | 180/210 | 85,7 | 0.81(0.52-1.25) | 0,333 | 0.82 (0.52-1.32) | 0,419 |  |
| **Grouped Generalized self-efficacy 2018** |  |  |  |  |  |  |  |
| Low self efficacy | 653/735 | 88,8 | 1 |  | 1 |  |  |
| High self efficacy | 374/436 | 85,8 | 0.76(0.53-1.08) | 0,124 | 0.80 (0.54-1.18) | 0,264 |  |
|  |  |  |  |  |  |  |  |
