# Supplementary material for: Impact of the DREAMS interventions on Common mental disorders among adolescent girls and young women: Causal analysis of a prospective cohort in two Kenyan settings
Source: PLOS Ment Health. 2026 Nov 3;3(11):e0000765. doi: 10.1371/journal.pmen.0000765 (PMC13730345; doi:10.1371/journal.pmen.0000765)
Supplement: Supplementary file 3 [file pmen.0000765.s003.docx]

Zahra Al-Khateeb, Ph.D
Staff Editor
PLOS Global Public Health

Re: Manuscript PGPH-D-25-00080

Impact of the DREAMS interventions on Common mental disorders among adolescent girls and young women: Causal analysis of a prospective cohort in two Kenyan settings

Thank you for sharing the peer reviews of our manuscript and inviting us to submit a revision. We are pleased that you and the reviewers saw the value of this work and appreciate their feedback.

We have responded to each comment below and uploaded revised versions (clean and with tracked changes) for your consideration.

We look forward to your response.

Sincerely,

Faith Magut, on behalf of the authors

Reviewer #1: This study addresses an important question using rigorous methods and large, well-characterized cohorts. However, the counterintuitive findings (higher CMD among DREAMS invitees) raise serious questions about causal inference. The authors' explanation of increased awareness/reporting is plausible but not definitively proven. The study would benefit from more conservative interpretation of findings and stronger acknowledgment of the limitations inherent in the quasi-experimental design. I have provided for the authors the specific changes required to strengthen the paper.

INTRODUCTION SECTION
Required Changes:
1.1 Strengthen Theoretical Framework

Issue: The paper lacks a clear conceptual model explaining HOW DREAMS interventions would impact mental health outcomes.
Action Required: Develop and present a logic model or conceptual framework showing the hypothesized pathways from DREAMS components (social asset building, mentorship, etc.) to CMD outcomes. Include this as a figure.
Specific Request: Add 2-3 paragraphs explicitly describing the theoretical mechanisms linking each DREAMS component to mental health.

We have developed a conceptual framework (now included as Figure 1 in the revised manuscript) illustrating the hypothesized pathways through which the DREAMS interventions may influence CMD among AGYW. The framework draws on existing literature linking social support, social protection, parent and caregiver programs, and health service access to mental health outcomes.

We have also included an expanded section in the Introduction (Line 128-158, page 5-6) describing in detail how specific DREAMS interventions including social asset building, financial capability, HIV prevention education, and community-level norm change, parent and caregiver program may affect CMDs through intermediate factors such as enhanced self-efficacy, improved social support, reduced exposure to violence, and decreased economic stress and improved relationships with parents/guardians.

1.2 Clarify Research Objectives

Issue: Current objective is vague ("investigated whether DREAMS interventions had an impact").
Action Required: Reformulate as specific, testable hypotheses. For example:

"We hypothesized that DREAMS interventions would reduce CMD prevalence among AGYW through increased social support and self-efficacy."
State primary and secondary objectives clearly.

Thank you for the suggestion. We have revised the objective to make it more specific and testable. We now explicitly state our primary and secondary objectives and include a clear hypothesis and revised text in the introduction (line 169-171, page 6) that, we aimed to estimate the prevalence of CMD among AGYW in two Kenyan settings, and aimed to examine whether exposure to DREAMS interventions was associated with a reduced prevalence of CMD.

METHODS SECTION
Required Changes:
2.1 Exposure Definition

Issue: Using "invitation to DREAMS" rather than actual participation may introduce misclassification bias.
Action Required:

Justify why invitation was chosen over participation

DREAMS was a programme implemented in “real-world” conditions, and not a randomised controlled trial, there was not a randomly-assigned control group to compare with a randomly-assigned intervention group. We used "invitation to DREAMS" rather than actual participation because our aim was to assess the real-world effectiveness of DREAMS.

“Invitation to DREAMS” is a well-defined and well understood by AGYW. Using invitation allows us to avoid potential bias that could arise from conditioning on participation since those who participate may differ systematically from those who do not. This approach is conceptually analogous to an intention-to-treat (ITT) analysis in randomised trials, where outcomes are analysed based on initial assignment rather than actual treatment received. We believe this provides a more relevant estimate of the impact of DREAMS as implemented in practice. We have clarified in Line 279-283, page 10.

Report participation rates among invitees
Conduct sensitivity analysis using actual participation as exposure

We considered the intention-to-treat (ITT) analysis to be the most appropriate and we do not wish to conduct a sensitivity analysis using actual participation as exposure (add). While we acknowledge that actual participation may vary, our aim is to estimate the population-level impact of DREAMS as implemented in practice in real world settings. A high proportion of DREAMS invitees received the DREAMS interventions. For example, participation in DREAMS interventions among invitees was high with around 70% receiving ≥3 primary package interventions by 2019 (Gourlay, A., et al, 2019). By 2022, In Gem, 97% among early DREAMS invitees and 88% among later invitees received ≥3 and in Nairobi 93% among early DREAMS invitees and 86% among later invitees received ≥3 by 2022 (paper in preparation).

Discuss potential impact of misclassification on results
We expect this misclassification very low, invitation to DREAMS activities was formalized and coordinated by a single implementing partner, providing a consistent and structured process in both study settings. AGYW will have understood well whether they had been invited to DREAMS (“DREAMS invitee”) or had not been invited to DREAMS (“non invitees”). We have included this in the limitation section in (Line 670-687, page 29)

2.2 Sample Size Justification

Issue: No power calculation provided.
Action Required: Add power calculation showing ability to detect clinically meaningful differences in CMD prevalence between groups.

The study sample size is described in detail in the published protocol paper (Birdthistle, et al,2018). In brief, for the nested cohorts in the HDSS sites in Kenya, a minimum of 400 adolescent girls and young women (AGYW) in each of the 13–17 and 18–22 year age groups was selected from the HDSS sampling frames. This sample size was chosen to enable the analysis of causal relationships between uptake of DREAMS interventions and key outcomes. To account for non-response and loss-to-follow-up, we oversampled by 20%. We have provided a reference to the protocol paper in the paper (Line 192, page 7)

2.3 Enhanced Propensity Score Diagnostics

Issue: Limited assessment of IPTW assumptions.
Action Required:

Provide standardized mean differences (SMD) tables before and after weighting

Thank you for the suggestion. We had done this but had not included in our paper but we have now included plots of standardized mean differences (SMDs) before and after weighting in the supplementary materials (see Supplementary Figures S2 Fig3a and S2 Fig3b, respectively).
Add supplementary figures showing propensity score distributions

We had already included the propensity score distributions in the supplementary materials . These can be found in S2 Fig1a and S2 Fig1b respectively

Test positivity assumption more rigorously

We assessed the positivity (overlap) assumption by examining the distribution of estimated propensity scores across exposure groups. Specifically, we plotted the distribution of propensity scores comparing DREAMS-invitees by 2018(early invitees) and non-invitees and DREAMS-invitees by 2022(later invitees) and non-invitees. We observed sufficient overlap between the two groups, indicating that the positivity assumption was satisfied. This is presented in Supplementary Figure S2 Fig1a and S2 Fig1b

In addition, we identified the proportion of observations with extreme propensity scores (e.g. <0.2 or >0.9) to evaluate potential violations of positivity. These were found to be minimal (0.06% in Nairobi & 0% in Gem), suggesting that very few individuals had zero probability of being in either group. We updated this in the methods section. Those with <0.2 in Nairobi were excluded from the analysis (Line 353-361, page 12)

Report extreme weights and their impact

We have now included the distribution of the stabilized inverse probability of treatment weights (IPTWs) in the supplementary materials (see Supplementary Figure S2 Fig3a and Fig3b). The figure shows the distribution of the weights. To address the potential influence of extreme weights on the estimates, we applied weight truncation at the 99th percentile (weights >3), as described in the Methods section in Line 405, page 14. The results with and without truncation were very similar, showing that our findings were robust to the presence of extreme weights.

2.4 Confounding Variables

Issue: DAG construction and variable selection poorly justified.
Action Required:

Move DAG to main text with detailed explanation

We have now moved the Directed Acyclic Graph (DAG) from the supplementary materials to the main manuscript (now included as Figure 2).

Justify each confounder's inclusion based on literature/expert knowledge

The directed acyclic graph (DAG) describing the hypothetical causal pathways considered for this study is shown in Figure 2. Age was conceptualized to influence education, pregnancy, sexual history, socio-economic status (SES), food insecurity, and access to credit, given that older AGYW are more likely to have sexual experience, earn income, and access finances, while younger AGYW are more likely to still be in school. Parental education was considered to shape SES, food insecurity, and access to credit through increased knowledge and earning, which in turn support educational attainment. Household SES was hypothesized to influence education by determining the ability to afford school fees and materials. Orphanhood was considered to affect education, SES, and food insecurity through the loss of parental support, while also increasing vulnerability to early pregnancy and risky sexual behaviours. Pregnancy and sexual history were further hypothesized to affect education through increased likelihood of school drop-out. We have included justification of the confounders inclusion (Line 290-300, page 11).

Discuss potential unmeasured confounders more thoroughly

Unmeasured factors for example marital status (measured in 2019 but not 2018 in Gem) may be associated both with invitation to participate in DREAMS and CMD. To guide confounder selection and reduce the risk of bias from unmeasured confounding, we constructed a Directed Acyclic Graph (DAG) informed by expert knowledge and existing literature. While the use of a DAG helps to identify potential confounders, the extent to which bias is minimized ultimately depends on the magnitude of unmeasured confounding and the degree to which these unmeasured factors are associated with both exposure and outcome. Presence of unmeasured confounders could result in an overestimation or underestimation of the association between DREAMS invitation and CMD. We have updated this in the limitation section of the paper. (Line 652-659, page 28)

RESULTS SECTION
Required Changes:
4.1 Effect Size Interpretation

Issue: Limited discussion of clinical significance.
Action Required:

Interpret effect sizes in clinical context
Compare to minimal clinically important differences from literature

The proportion of AGYW with CMD was around 10% higher among DREAMS invitees, which is clinically meaningful. Such increase may reflect improved mental health literacy due to the DREAMS intervention, leading to greater symptom recognition and willingness to disclose mental health problems rather than an actual worsening of CMD prevalence. This is critical from a public health perspective because it identifies previously unmet mental health needs and potentially better long-term outcomes. Therefore, while these findings do not suggest that DREAMS increases CMD causally, they highlight the intervention’s role in identifying mental health issues (Line 630-634, page 27).

Discuss number needed to harm if applicable
We think is not applicable and so have not attempted to calculate this.

4.2 Subgroup Analyses

Issue: Age-stratified results have wide confidence intervals suggesting insufficient power.
Action Required:

Acknowledge power limitations in subgroup analyses
Consider combining age groups if power is insufficient

We have presented an overall analysis and the subgroup analysis by age is a secondary analysis. While we explored differences in the impact of DREAMS by age group (younger vs older AGYW), the resulting estimates had wide confidence intervals, reflecting limited precision as highlighted in Line 507-510 Page 25. Although age stratification was conceptually important to assess whether the impact varied between early adolescence (13–17 years) and young adulthood (18–22 years), the limited statistical power constrains the strength of conclusions that can be drawn. However, we believe that presenting these findings is valuable, as they offer important insights into age-specific effects. The DREAMS programme uses a layered approach, with a defined “primary package” of core services tailored to specific age bands (10–14, 15–19, and 20–24 years), making it essential to consider age when evaluating programme impact.

Report interaction tests between age and intervention
We have reported the interaction test p values (Line 491-492, 502-503, page 21-22)

4.3 Temporal Trends

Issue: CMD increase over time inadequately explained.
Action Required:

Provide more detailed analysis of temporal changes

Thank you for comment regarding the analysis of temporal changes. While the dataset would allow for exploration of changes in CMD prevalence over time, the focus of this paper was on estimating overall prevalence of CMD and assessing the impact of DREAMS on CMD by comparing invitees and non-invitees. Temporal trends were therefore not included in the analyses presented here.

.
Discuss COVID-19 impact explicitly

We have discussed the COVID-19 impact explicitly in (Line 588-596, page 25-26) that a study in Kenya showed that approximately 30% of young women reported symptoms consistent with probable depression during the pandemic, while qualitative evidence linked these elevated rates directly to pandemic-related isolation, economic hardship, and disruptions to daily life(Gichangi, P.B., et al, 2023). Additionally, findings from the study confirm that the challenges posed by COVID-19 such as school closures, exposure to violence, and socioeconomic stressors contributed to a rise in mental health issues, especially among adolescent girls. Therefore, these pandemic-related stressors may have contributed to the rise in CMD over time, independent of DREAMS exposure.

5.2 Alternative Explanations

Issue: Residual confounding and selection bias inadequately addressed.
Action Required: Expand discussion of:

How unmeasured confounders could explain findings

Despite efforts to adjust for measured confounders using inverse probability of treatment weighting (IPTW), the possibility of residual confounding cannot be ruled out. Unmeasured factors for example marital status may be associated both with invitation to participate in DREAMS and CMD. To guide confounder selection and reduce the risk of bias from unmeasured confounding, we constructed a Directed Acyclic Graph (DAG) informed by expert knowledge and existing literature. Presence of unmeasured confounders could result in an overestimation or underestimation of the association between DREAMS invitation and CMD. However, However, we attempted to measure and control for known individual and household characteristics that were used by implementing partners to guide who was invited to DREAMS. We have discussed the impact of unmeasured confounders in (Line 622-629 , Page 29)

Potential for reverse causation

Our study was designed to estimate the impact of DREAMS invitation on CMD outcomes, we clearly defined the temporal relationship between exposure and outcome. AGYW were invited to participate in DREAMS based on socio-economic and sexual risk characteristics but not an assessment of mental health. We have updated this in the limitation section of the paper (Line 652-659, page 28)

Selection bias from non-random invitation process

In our study, the invitation to participate in the DREAMS intervention was not randomised but instead guided by programmatic criteria designed to identify AGYW based on a socio-economic and sexual risk vulnerability criteria . This non-random invitation process may have introduced systematic differences between those who were invited and those who were not. For instance, DREAMS invitees differed in key socio-demographic characteristics e.g. food insecurity, SES, behavioural characteristics eg sexual and pregnancy history, and these factors influenced CMD. To address this, we used inverse probability of treatment weighting (IPTW) to balance characteristics between the invitation groups. However, given the observational nature of the study, we acknowledge that residual selection bias cannot be entirely ruled out. However, we believe it is unlikely to have substantially influenced our findings, given the steps taken to adjust for measured confounders (Line 670-692 page 29)

5.3 Mechanistic Interpretation

Issue: The "increased awareness" explanation is plausible but not proven.
Action Required:

Present this as one of several possible explanations
Suggest how this hypothesis could be tested
Discuss implications if this explanation is correct

DREAMS may have created a supportive environment that helped AGYW better recognize and articulate their mental health challenges, they might otherwise conceal due to shame, fear, or social pressure through mentorship, safe spaces, and life skills sessions,. Participation in DREAMS may also foster trusting relationships with mentors, which could further encourage openness and disclosure of poor mental health indicators. To better understand this mechanism, qualitative research is needed to explore AGYW’s perceptions of mental health and how DREAMS may shape their recognition and reporting of symptoms. However, the consistent pattern of higher CMD prevalence among both early and later invitees, across both Gem and Nairobi, supports the interpretation that increased awareness and willingness to report symptoms may be a key mechanism rather than chance or site-specific effects. We have expanded on this in the discussion section by adding additional interpretation (line 565-569 page 25)

5.4 Clinical and Policy Implications

Issue: Recommendations are vague and not actionable.
Action Required:

Provide specific recommendations for program implementers
Discuss cost implications of adding mental health components
Address feasibility and scalability concerns

We have now provided specific recommendations for program implementers, discussed cost implications and addressed feasibility and scalability concerns. As indicated in Line 728-745, page 30-31,we recommend integrating mental health services (for example, cognitive behavioral therapy (CBT)-based group sessions) and peer support into the DREAMS package, delivered through existing safe spaces. DREAMS mentors should be trained in mental health literacy, identification of poor mental health indicators, and appropriate referral for mental health support. While this will incur additional costs, these could be modest and might result in substantial benefits, including improved well-being and improved HIV prevention outcomes. Moreover, integration is feasible through gradual adaptation and can be scaled by utilising existing infrastructure, like schools and safe spaces.

6. LIMITATIONS SECTION
Required Changes:
6.1 Expand Limitations Discussion

Action Required: Add discussion of:

Measurement error in both exposure and outcome

There is potential measurement error in both the exposure and outcome variables. The exposure invitation to participate in DREAMS was self-reported which may be subject to recall bias or misclassification. Similarly, the outcome symptoms of common mental disorders (CMD) was based on self-reported screening tools rather than clinical diagnoses, which may lead to under- or overestimation of true CMD prevalence. We have updated this in the limitation section (line 670-687, page 29)

Potential spillover effects to non-invitees

We appreciate the comment regarding potential spillover effects. In our study, we assumed no interference between individuals (i.e., that each AGYW’s outcomes were independent of others’ treatment status), consistent with the Stable Unit Treatment Value Assumption (SUTVA). We believe this assumption is generally reasonable, as our primary outcome, CMD is largely influenced by individual experiences and structural vulnerabilities. While it is possible that spillover may have occurred through shared knowledge, peer influence, or exposure to community-level components of DREAMS, especially among AGYW living in the same household or community, we expect such instances to be limited. If present, such spillover would likely bias the estimated effects toward the null, making our findings conservative. We have updated this in the limitation section (line 693-698 page 29)

Competition from other programs

We think the competition from other programs was minimal because DREAMS was the primary multi-sectoral intervention targeting AGYW in the study settings during the study period. The other programs focused on provision of services and there was limited social support programs. We have updated this in the Limitation section in (Line 699-711, page 30)

Generalizability limitations beyond Kenya

We have updated limitations to the generalizability of our findings in the limitation section (Line 717-721, page 30). We have stated that although our study was conducted in two Kenyan settings, the findings are likely to be broadly generalisable to similar DREAMS implementation contexts in East Africa. However, our results may not be generalisable to all countries where DREAMS is implemented, but because they represent diverse implementation contexts they can still contribute important insights for other settings in which DREAMS (or other combination HIV prevention) interventions are being implemented.

6.2 Impact on Interpretation

Action Required: For each limitation, discuss how it might affect results and conclusions.

We have made these revisions suggested (page 29-30, line 652-721).

SPECIFIC QUESTIONS FOR AUTHORS

Why was invitation chosen over actual participation as the exposure? Please provide stronger justification or consider alternative exposure definitions.

How do you rule out reverse causation? Could existing mental health issues make AGYW more likely to be invited to DREAMS?

Our study was designed to estimate the impact of DREAMS invitation on CMD outcomes, we clearly defined the temporal relationship between exposure and outcome. AGYW were invited to participate in DREAMS based on socio-economic and sexual risk vulnerability criteria. The invitation process was not random, but based on factors such as being pregnant, being of school age but out of school, experiencing food insecurity or household poverty, or being an orphan. Although we clearly defined “invitation to DREAMS” as our exposure, the reliance on socio-economic and sexual risk vulnerability criteria may introduce the possibility that pre-existing CMD symptoms may have influenced eligibility for DREAMS. However, this is unlikely given that invitations were primarily based on predefined socio-economic and sexual risk vulnerability criteria rather than mental health status. We have updated this in the limitation section of the paper in Line 688-692, page 29.

What is the clinical significance of the observed differences? How do effect sizes compare to minimal clinically important differences?

The proportion of AGYW with CMD was around 10% higher among DREAMS invitees, which is clinically meaningful. Such increase may reflect improved mental health literacy due to the DREAMS intervention, leading to greater symptom recognition and willingness to disclose mental health problems rather than an actual worsening of CMD prevalence. This is important from a public health perspective because it identifies previously unmet mental health needs and potentially better long-term outcomes. Therefore, while these findings do not suggest that DREAMS increases CMD causally, they highlight the intervention’s role in identifying mental health issues (Line 630-634, page 27)

How confident are you in the "increased awareness" explanation? What evidence supports this interpretation over residual confounding?

The higher CMD prevalence among both early and later invitees, across both Gem and Nairobi, supports the interpretation that increased awareness and willingness to report symptoms may be a key mechanism rather than chance or site-specific effects. DREAMS includes components such as safe spaces, mentorship, and life skills training all of which may improve mental health literacy. If residual confounding were the primary driver, we might expect inconsistent patterns or associations limited to early invitees or one site. We have given possible explanations to our findings in line 563-569, page 25)

What are the practical implications? How would mental health components be integrated into DREAMS programming?

We recommend integrating mental health services such as cognitive behavioral therapy (CBT)-based group sessions and peer support into the DREAMS package, delivered through existing safe spaces. DREAMS mentors should be trained in mental health literacy, identification of poor mental health indicators, and appropriate referral to support services (Line 728-745, page 31).

6. PLOS authors have the option to publish the peer review history of their article ([what does this mean?](https://track.editorialmanager.com/CL0/https:%2F%2Fjournals.plos.org%2Fglobalpublichealth%2Fs%2Feditorial-and-peer-review-process%23loc-peer-review-history/1/010f019807afa245-56545f9c-ca73-4ea7-b745-8eb786a9c7e5-000000/L6Sw0FfjPIUXwKer3BS6TeD2CElNPM6icTp0SXrDdsM=219)). If published, this will include your full peer review and any attached files.

Yes
**Do you want your identity to be public for this peer review?** If you choose “no”, your identity will remain anonymous but your review may still be made public.


For information about this choice, including consent withdrawal, please see our [Privacy Policy](https://track.editorialmanager.com/CL0/https:%2F%2Fwww.plos.org%2Fprivacy-policy/1/010f019807afa245-56545f9c-ca73-4ea7-b745-8eb786a9c7e5-000000/U7wT9BfeJyA8ZmjciQvr23hym7uMaD6WH8oDFmUTy9E=219).

Reviewer #1: No
