# Supplementary material for: Impact of the DREAMS interventions on Common mental disorders among adolescent girls and young women: Causal analysis of a prospective cohort in two Kenyan settings
Source: PLOS Ment Health. 2026 Nov 3;3(11):e0000765. doi: 10.1371/journal.pmen.0000765 (PMC13730345; doi:10.1371/journal.pmen.0000765)
Supplement: Supplementary file 4 [file pmen.0000765.s004.docx]

CC: [andreas.haas@unibe.ch](mailto:andreas.haas@unibe.ch)

PMEN-D-26-00057
Impact of the DREAMS interventions on Common mental disorders among adolescent girls and young women: Causal analysis of a prospective cohort in two Kenyan settings
PLOS Mental Health

Dear Dr. Magut,

Thank you for submitting your manuscript to PLOS Mental Health. After careful consideration, we feel that it has merit but does not fully meet PLOS Mental Health’s publication criteria as it currently stands. Therefore, we invite you to submit a revised version of the manuscript that addresses the points raised during the review process.

Please submit your revised manuscript by Jul 18 2026 11:59PM. If you will need more time than this to complete your revisions, please reply to this message or contact the journal office at [mentalhealth@plos.org](mailto:mentalhealth@plos.org). When you're ready to submit your revision, log on to <https://www.editorialmanager.com/pmen/> and select the 'Submissions Needing Revision' folder to locate your manuscript file.

Please include the following items when submitting your revised manuscript:

- A letter that responds to each point raised by the editor and reviewer(s). You should upload this letter as a separate file labeled 'Response to Reviewers'.
- A marked-up copy of your manuscript that highlights changes made to the original version. You should upload this as a separate file labeled 'Revised Manuscript with Track Changes'.
- An unmarked version of your revised paper without tracked changes. You should upload this as a separate file labeled 'Manuscript'.

Guidelines for resubmitting your figure files are available below the reviewer comments at the end of this letter.

As the corresponding author, your ORCID iD is verified in the submission system and will appear in the published article. PLOS supports the use of ORCID, and we encourage all coauthors to register for an ORCID iD and use it as well. Please encourage your coauthors to verify their ORCID iD within the submission system before final acceptance, as unverified ORCID iDs will not appear in the published article. *Only* the individual author can complete the verification step; PLOS staff *cannot* verify ORCID iDs on behalf of authors.

We look forward to receiving your revised manuscript.

Kind regards,

Andreas Haas
Academic Editor
PLOS Mental Health

**Journal Requirements:**

1. Please include a complete copy of PLOS’ questionnaire on inclusivity in global research in your revised manuscript. Our policy for research in this area aims to improve transparency in the reporting of research performed outside of researchers’ own country or community. The policy applies to researchers who have travelled to a different country to conduct research, research with Indigenous populations or their lands, and research on cultural artefacts. The questionnaire can also be requested at the journal’s discretion for any other submissions, even if these conditions are not met.  Please find more information on the policy and a link to download a blank copy of the questionnaire here: [https://journals.plos.org/mentalhealth/s/best-practices-in-research-reporting](https://url.uk.m.mimecastprotect.com/s/dvjlCM1Gzc3k29AIwf8U8TZt6?domain=journals.plos.org). Please upload a completed version of your questionnaire as Supporting Information when you resubmit your manuscript.

2. Thank you for uploading your study's underlying data set. Unfortunately, the repository you have noted in your Data Availability statement does not qualify as an acceptable data repository according to PLOS's standards.
 
 At this time, please upload the minimal data set necessary to replicate your study's findings to a stable, public repository (such as figshare or Dryad) and provide us with the relevant URLs, DOIs, or accession numbers that may be used to access these data. For a list of recommended repositories and additional information on PLOS standards for data deposition, please see [https://journals.plos.org/plosone/s/recommended-repositories](https://url.uk.m.mimecastprotect.com/s/6ajbCN0JAi3PZ9GI4hzUyiKno?domain=journals.plos.org).

3. Please ensure that your Ethics Statement is available in its entirety at the beginning of your Methods section, under a subheading 'Ethics Statement'.

If the reviewer comments include a recommendation to cite specific previously published works, please review and evaluate these publications to determine whether they are relevant and should be cited. There is no requirement to cite these works unless the editor has indicated otherwise. 

**Additional Editor Comments (if provided):**

 The study addresses an important question and uses methods intended to support causal interpretation. I have concerns about the manuscript’s somewhat counterintuitive finding that the DREAMS intervention led to higher CMD symptoms. This finding requires careful scrutiny and explanation.

The reviewers considered the study to be generally well conducted. However, they identified several issues that require clarification and several methodological concerns that require substantial revision. In particular, the revised manuscript should address the following points:

1. The manuscript should provide a clearer and more detailed description of the eligibility criteria for the DREAMS intervention and the invitation process.
2. Please clarify the causal estimand.
3. The reviewers raised concerns about residual confounding. The revised manuscript should explain whether the available covariates adequately capture the factors that influenced DREAMS invitation. Baseline CMD levels may be especially relevant if they were associated with factors that influenced invitation. Please discuss whether baseline CMD can be included in the adjustment set and, if not, why not.
4. Please expand the discussion of the counterintuitive main finding in line with the reviewers’ recommendations.
5. Please provide more detail on censoring, the assumed censoring mechanism, and the use of inverse probability of censoring weights. Consider using a DAG.
6. Please clarify the intervention components that may affect mental health.

I invite you to submit a major revision. Please submit a point-by-point response to all reviewer comments and revise the manuscript accordingly. The revised manuscript will likely be returned to the reviewers for further assessment.


[Note: HTML markup is below. Please do not edit.]

Reviewers' comments:

Reviewer's Responses to Questions

**Comments to the Author**

1. Does this manuscript meet PLOS Mental Health’s [publication criteria](https://url.uk.m.mimecastprotect.com/s/vKxNCO8KBCxZNwyfkirUGpm9j?domain=track.editorialmanager.com)? Is the manuscript technically sound, and do the data support the conclusions? The manuscript must describe methodologically and ethically rigorous research with conclusions that are appropriately drawn based on the data presented.

Reviewer #1: Yes

Reviewer #2: Yes

2. Has the statistical analysis been performed appropriately and rigorously?

Reviewer #1: Yes

Reviewer #2: Yes

3. Have the authors made all data underlying the findings in their manuscript fully available (please refer to the Data Availability Statement at the start of the manuscript PDF file)?

The [PLOS Data policy](https://url.uk.m.mimecastprotect.com/s/JwB5CPQLDUXN0ZkhjsgUx_6Cp?domain=track.editorialmanager.com) requires authors to make all data underlying the findings described in their manuscript fully available without restriction, with rare exception. The data should be provided as part of the manuscript or its supporting information, or deposited to a public repository. For example, in addition to summary statistics, the data points behind means, medians and variance measures should be available. If there are restrictions on publicly sharing data—e.g. participant privacy or use of data from a third party—those must be specified.

Reviewer #1: No

Reviewer #2: Yes

4. Is the manuscript presented in an intelligible fashion and written in standard English?

PLOS Mental Health does not copyedit accepted manuscripts, so the language in submitted articles must be clear, correct, and unambiguous. Any typographical or grammatical errors should be corrected at revision, so please note any specific errors here.

Reviewer #1: Yes

Reviewer #2: Yes

5. Review Comments to the Author

Please use the space provided to explain your answers to the questions above. You may also include additional comments for the author, including concerns about dual publication, research ethics, or publication ethics. (Please upload your review as an attachment if it exceeds 20,000 characters)

**Reviewer #1:** The current manuscript evaluates the impact of *offering* the DREAMS interventions package on a secondary outcome, mental disorders. Overall, I find the paper to be well written and of decent methodological quality (and appropriate diagnostics). The critical part are the results and their interpretation (though I do not see any problem of reporting a “negative result”). The authors have offered some angles around that, but I suggest further disentangling in this regard. What could explain the results? I think there are at least the following options:

a) Measurement error in the outcome, due to the self-reporting of CMD. Discussed by the authors and speculated to be relevant.

CMD was assessed using a validated self-reported instrument; and such measures could be inherently subject to misclassification, including reporting bias and variation in individual interpretation of symptoms. If this misclassification is non-differential with respect to DREAMS invitation, it would be expected to attenuate any true effect towards the null, potentially contributing to the absence of a clear association.

However, we think that measurement error alone is less likely to fully explains the observed patterns, particularly given that the instrument has been widely used in similar settings and the CMD prevalence estimates are consistent with those reported in other studies in similar settings. Additionally, our data show clear variation in CMD across groups (e.g., by age group) suggesting the instrument is sensitive to contextual differences.

We have update the limitation section in Page 30 , line 648-654 that: *CMD was measured using the self-reported Shona Symptom Questionnaire and may therefore be subject to outcome misclassification due to reporting bias or differences in participants' interpretation of symptoms. If such misclassification was non-differential with respect to DREAMS invitation, it would be expected to attenuate the estimated associations towards the null. However, the Shona Symptom Questionnaire has been widely validated in similar settings, and the variation in CMD prevalence observed across participant characteristics in our study suggests that the instrument was able to differentiate between groups with differing levels of CMD*.

b) An actual small negative effect. Appropriately discussed by the authors.

Thank you for the feedback

c) The ITT approach. Partly discussed. I agree that from a policy perspective “offering DREAMS” can be seen as a primary target. That’s fine. I still think there is no way around describing in more detail who, and how many participants, who were offered DREAMS accepted this. A potential explanation of the results is that there is a (small) benefit for the population who accepted DREAMS (only plausible pathway); and unmeasured residual confounding (see below) for the “vulnerable” group (with poorer outcomes) who did not. At a minimum the authors could describe proportion accepting the interventions and logistic models to describe who agreed to participate (at the different stages). A (encouraged) bonus would be using this information for reporting a short result on the effect of accepted DREAMS on the outcome (in the supplementary material), not to replace the initial analysis but to support the final interpretation of the primary results.

We used "invitation to DREAMS" rather than actual participation because our aim was to assess the real-world effectiveness of DREAMS. “Invitation to DREAMS” is a well-defined and well understood by AGYW. Using invitation allows us to avoid potential bias that could arise from conditioning on participation since those who participate may differ systematically from those who do not.

We acknowledge that analyses based on actual participation could provide additional insight. However, as participation is not randomly assigned, the analyses could be subject to selection bias and residual confounding, given that uptake may be related to underlying vulnerability and other unmeasured factors that also influence mental health outcomes.

We have data from other papers in the same project which show that participation in DREAMS interventions among invitees was high. In page 10 line 255-256, we state that, *approximately 70% of invitees received ≥3 primary package interventions by 2019 (Gourlay et al., 2019).* By 2022, uptake remained high, with 97% of early invitees and 88% of later invitees in Gem, and 93% of early invitees and 86% of later invitees in Nairobi, receiving ≥3 primary interventions (paper in progress). These high levels of uptake suggest that the ITT estimates are unlikely to be substantially attenuated due to non-participation

d) Unmeasured confounding. A strength of the study is the intense measuring of variables. However, treatment assignment/offering was based on the Girl Roster census method, whereas adjustment is based on “proxy variables”. I suggest the authors are very precise on the difference between them and explain to what degree they are different, to reflect on unmeasured confounding. Also, to what degree can one adjust for this “Girl Roster” method? Additionally, it would be helpful to see somewhere the logistic models for the probability of being offered (early/late) DREAMS, to understand who got it.

The Girl Roster method represents a structured approach to identifying eligible AGYW based on multiple dimensions of vulnerability, including schooling status, sexual activity, orphanhood, and other socio-economic characteristics. While we do not directly observe the full decision process used in programme implementation, many of the variables included in our adjustment set (e.g., age, education, pregnancy history, sexual behaviour, and socio-economic indicators) are intended to capture these same domains that are used in the roster. As such, our adjustment can be viewed as approximating key components of the Girl Roster criteria, although not fully replicating it. We included a comprehensive set of covariates in our adjustment and therefore expect residual confounding to be limited. However, unmeasured or imperfectly measured factors cannot be ruled out.

In the analysis, confounding was addressed using inverse probability of treatment weighting (IPTW) which is based on the propensity score approach, defined as the probability of invitation to DREAMS conditional on the set of covariates (adjustment set). The purpose of the approach is to achieve balance in the distribution of measured confounders between comparison groups. We think that inclusion of the results from the propensity score model is not necessary for the primary analysis, provided that adequate covariate balance is achieved, which we assessed using standard diagnostic measures and we have presented these diagnostic measures in the supplementary materials.

We have acknowledged this in the limitation sections page 29, line 599-602 that : *Although we adjusted for a comprehensive set of characteristics, these variables may not fully capture the context-specific criteria used in the Girl Roster method for invitation to DREAMS. As a result, residual confounding due to unmeasured factors cannot be ruled out.*

e) Time. It is CMD in 2022. Maybe there would be an effect if measured earlier, e.g shortly after the package was received. Can the authors comment on descriptive data being available for this and the potential impact?

We agree that the timing of outcome measurement may influence the ability to observe an effect of DREAMS on CMD. We explored earlier CMD prevalence descriptively, as shown in Figure 4.

Our primary analysis focused on CMD measured in 2022. For early invitees, this reflects a longer-term outcome following invitation. In contrast, for later invitees, CMD was measured relatively soon after invitation and therefore reflects outcomes over a shorter follow-up period. Despite this, we observed no evidence of a reduction in CMD among later invitees. We acknowledge that short-term effects among early invitees cannot be ruled out. However, DREAMS was designed to address multiple structural, social, and behavioural determinants of health, many of which may require sustained exposure before translating into measurable improvements in mental health. Consequently, any effects on CMD may be gradual rather than immediate.

We have updated the manuscript page 30-31 line 655-661 that : *A further consideration is the timing of the outcome assessment. CMD was measured in 2022, which for later invitees was relatively soon after invitation and so reflects a more immediate outcome. While we found no evidence of a reduction in CMD among later invitees, for early invitees we cannot rule out the possibility that there were short-term effects that had attenuated by the time of the outcome assessment in 2022. However, DREAMS was designed to address multiple structural, social, and behavioural determinants of health, and it was intended to have lasting effects on the AGYW who participated in the programme.*

f) Censoring. The authors used IPCW. The respective model could be reported, similar to the point raised under d). The possible censoring mechanism should be added to the DAG . Could there be unmeasured confounding with respect to drop-out?

The IPCW model included characteristics that were identified as strong predictors of loss to follow-up, including age, DREAMS invitation by 2018, pregnancy history, and sexual history. These variables were used to estimate each participant's probability of remaining under follow-up, from which the censoring weights were derived.

We have clarified the assumed censoring mechanism in the Methods page 14 line 359-360 that : *The IPCW approach assumes that, conditional on these characteristics included in the censoring model, loss to follow-up is independent of the potential CMD outcomes.*

We have now added information on the censoring model in the supplementary material (S2 Table 4a and S2 Table 4b). We acknowledge that unmeasured predictors of loss to follow-up may remain and, if these are also associated with CMD, residual selection bias due to informative censoring cannot be ruled out.

This limitation has now been acknowledged in the Discussion page 29 line 635-638 that: *Although we used inverse probability of censoring weighting to account for differential loss to follow-up, and we adjusted for a set of characteristics associated with follow-up, unmeasured predictors of loss to follow-up may remain and could result in residual selection bias.*

We did not include a directed acyclic graph (DAG) because the covariates included in the censoring models were selected a priori based on substantive knowledge rather than through a graphical causal model. We have instead clarified the assumptions underlying the censoring model in the Methods and provided the censoring model in the Supplementary Material.

g) Chance. Mayn hypothesized differences are consistent with data, given the assumptions. See also Sander Greenlands writings.

Throughout the paper, we present effect estimates together with their corresponding 95% confidence intervals, which quantify the uncertainty around the estimated effects arising from sampling variability under the assumptions of the analysis.

We have acknowledged this in the paper page 31, line 653-656 that:

*Finally, our findings should be interpreted considering random error. As with any study based on a sample rather than the entire population, the estimated effects are subject to sampling variability. Although we present 95% confidence intervals to quantify this uncertainty, the observed estimates remain compatible with a range of plausible effect sizes under the assumptions of the analysis.*

h) Assumptions violations. The reported diagnostics are however re-assuring in this respect.

Thank you for the feedback

My proposal is to work through these explanations, and do the above suggested small analyses, to then carefully re-visit conclusions and interpretations, as necessary.
As a side note: I find presenting results in Figures always nicer than in tables. The main results of the paper could be easily conveyed in that way.

Thank you for this suggestion. The main results are presented in both figures and tables. We have retained the tables because they provide the detailed numerical estimates compared to figures.

**Reviewer #2:** The manuscript is clearly structured and generally well written. My main concern relates to the findings, which appear somewhat counterintuitive. I suggest providing a more detailed description of the invitation process, including information on exactly who was eligible to be invited and how the selection criteria relate to the covariates included in the adjustment set.

I also recommend discussing baseline CMD levels across the observed comparison groups at the beginning of follow-up (2017–2019), and considering their inclusion in the adjustment set if appropriate. One might also reconsider the target estimand, for example by considering the average effect of invitation among those invited (i.e., an ATT with respect to invitation) as a complementary perspective. See detailed comments in the attached pdf.

6. PLOS authors have the option to publish the peer review history of their article ([what does this mean?](https://url.uk.m.mimecastprotect.com/s/ynnnCQ7METPlX9ytrtOUGqysb?domain=track.editorialmanager.com)). If published, this will include your full peer review and any attached files.


**Do you want your identity to be public for this peer review?** If you choose “no”, your identity will remain anonymous but your review may still be made public.


For information about this choice, including consent withdrawal, please see our [Privacy Policy](https://url.uk.m.mimecastprotect.com/s/oXf_CR1NGcW0GQzTouGU1ajIG?domain=track.editorialmanager.com).

Reviewer #1: No

Reviewer #2: No

[NOTE: If reviewer comments were submitted as an attachment file, they will be attached to this email and accessible via the submission site. Please log into your account, locate the manuscript record, and check for the action link "View Attachments". If this link does not appear, there are no attachment files.]

**Figure Resubmissions:**

While revising your submission, we strongly recommend that you use PLOS’s NAAS tool ([https://ngplosjournals.pagemajik.ai/artanalysis](https://url.uk.m.mimecastprotect.com/s/05LtCVQRMUyk2XRcWCBUEl-cf?domain=ngplosjournals.pagemajik.ai)) to test your figure files. NAAS can convert your figure files to the TIFF file type and meet basic requirements (such as print size, resolution), or provide you with a report on issues that do not meet our requirements and that NAAS cannot fix.

After uploading your figures to PLOS’s NAAS tool - [https://ngplosjournals.pagemajik.ai/artanalysis](https://url.uk.m.mimecastprotect.com/s/05LtCVQRMUyk2XRcWCBUEl-cf?domain=ngplosjournals.pagemajik.ai), NAAS will process the files provided and display the results in the "Uploaded Files" section of the page as the processing is complete. If the uploaded figures meet our requirements (or NAAS is able to fix the files to meet our requirements), the figure will be marked as "fixed" above. If NAAS is unable to fix the files, a red "failed" label will appear above. When NAAS has confirmed that the figure files meet our requirements, please download the file via the download option, and include these NAAS processed figure files when submitting your revised manuscript.

*In compliance with data protection regulations, you may request that we remove your personal registration details at any time.* [*(Remove my information/details)*](https://url.uk.m.mimecastprotect.com/s/nzOpCWqVNfqX6xWTLFYUoSFsS?domain=track.editorialmanager.com)*. Please contact the publication office if you have any questions.*

# **Reviewer 2**

First Review of manuscript PMEN-D-26-00057

Summary

The article examines associations between the Determined-Resilient-Empowered-AIDS-free-Mentored-Safe (DREAMS) intervention program targeted at a selection of adolescent girls and young women (AGYW) in two Kenyan settings and a measure of common mental health (CMD) at the end of follow-up, using the Shona Symptom Questionnaire.

The causal analysis considers hypothetical interventions on program invitation status, contrasting scenarios in which all AGYW are invited by 2018, all are invited at a later stage (2020–2022), or none are invited during the entire follow-up. The authors use an intention-to-treat estimand, corresponding to the effect of invitation, and implement inverse probability weighting for estimation.

The manuscript is clearly structured and generally well written. My main concern relates to the findings, which appear somewhat counterintuitive. I suggest providing a more detailed description of the invitation process, including information on exactly who was eligible to be invited and how the selection criteria relate to the covariates included in the adjustment

set. I also recommend discussing baseline CMD levels across the observed

comparison groups at the beginning of follow-up (2017–2019), and considering their inclusion in the adjustment set if appropriate. One might also reconsider the target estimand, for example by considering the average effect of invitation among those invited (i.e., an ATT with respect to invitation) as a complementary perspective.

Comments

• p. 2, Methods: The enrollment described here does not yet refer to the DREAMS program. Please clarify

We clarify that enrolment in the methods section refers to participation in the observational cohort, which was established independently of the DREAMS program. Eligibility for cohort enrolment was based on predefined study criteria and separately from DREAMS program implementation. The cohort was randomly selected from the general population of AGYW, and was enrolled in order to evaluate DREAMS. *We add “study” in page 7 line 176 to improve clarify.*

• Primary and secondary interventions: Are the primary and secondary interventions mentioned in the manuscript just examples (maybe the most important ones?) of a broader range of interventions that are assumed to promote better mental health outcomes? If not, list all interventions relevant to mental health, maybe in the appendix or in a table. In addition, clarify how access to non-mandatory, secondary interventions was determined, and which participants received these components.

Thank you for the comment.

The primary and secondary interventions described in the manuscript correspond to the components of the DREAMS package as defined in the study setting. We have referenced Birdthistle et al. (2018) (reference 20), which provides a comprehensive list of the primary and secondary interventions in Kenya. We explicitly specify the intervention components hypothesized to influence mental health outcomes in Figure 1. This includes both primary interventions, which were each AGYW was intended to receive, and secondary interventions, which were delivered based on individual need. We have also clarified how access to secondary (non-mandatory) interventions was determined. Access to secondary (non-mandatory) interventions was determined based on individual-level need and aimed at specific subgroups of AGYW according to their circumstances. This allocation was guided by assessments conducted by program implementers. For example, condom provision was targeted to AGYW who were sexually active, while post-violence care was provided to those who had experienced sexual violence.

We have added the following additional detail in page 5 , line 121-124 that: *The DREAMS package comprises interventions targeted towards individuals, wider communities, families, and AGYW partners divided into “must-have” primary interventions and secondary interventions delivered based on need (for example, condom provision for AGYW who are sexually active; post-violence care for AGYW who have experienced sexual violence)*

• It is not entirely clear who was selected to be eligible for the DREAMS program and who was ultimately invited. The manuscript would benefit from a more detailed description of the invitation process, including the criteria used to determine eligibility and invitation. In particular, it appears that the set of covariates used for confounding adjustment does not fully capture the factors underlying the invitation mechanism.

Clarify how the measured confounders relate to the criteria used in the selection process.

Thank you for this comment.

In the manuscript (Page 8, lines 207–212), we state that invitation to participate in DREAMS was targeted and not offered to all AGYW. Eligibility for invitation was determined based on socio-economic and sexual risk vulnerability, including factors such as pregnancy (ever or current), food insecurity, low household socioeconomic status, being out of school, and orphanhood. These characteristics were used to classify AGYW into risk profiles, and vulnerable individuals were identified using the “Girl Roster” census method.

Invitation to DREAMS was therefore not random but driven by these vulnerability assessments. As such, the invitation is related to individual-level characteristics that may also influence mental health outcomes. To clarify the relationship between the selection process and the adjustment set, we state in the Methods section in page 11, line 282-286, *These factors represent socio-economic and sexual risk vulnerability characteristics that informed eligibility and invitation to DREAMS and are plausibly associated with both the exposure (invitation to DREAMS) and the outcome (CMD).* For example, AGYW who were orphaned, had experienced pregnancy, or were from households with low socioeconomic status were prioritised for invitation, reflecting targeted programme delivery to higher-risk groups In addition, we refer to the directed acyclic graph (DAG) presented in Figure 2, which formalizes our assumptions about the relationships between invitation, measured confounders, and CMD, and guided the identification of an appropriate adjustment set.

• If invitation to DREAMS depended on deterministic rules, what does this mean with respect to the positivity assumption? Please clarify

We agree that if invitation to DREAMS were strictly determined by observed vulnerability criteria, this could raise concerns regarding violations of the positivity assumption. However, in practice, although invitation was guided by these criteria, it was not strictly deterministic, and variation in invitation status was observed within levels of these characteristics.

Implementation depended on programmatic factors such as resource constraints and operational challenges . As a result, not all AGYW meeting specific vulnerability criteria were invited, resulting in some variability in invitation. In addition, Table 1a and Table 1b shows the distribution of key characteristics across invited and non-invited groups, illustrating overlap in these factors and supporting the plausibility of the positivity assumption.

• Clarify whether individuals who were never invited to participate in the DREAMS program received any alternative services or interventions.

In page 10 line 257-259 we clarify that individuals who were not invited to participate in the DREAMS program were not excluded from accessing other services available in the community. These may have included standard health, social, or educational services provided outside of the DREAMS programme.

• Given that the DREAMS program appears to target a specific subgroup, clarify whether the estimand is intended to capture the effect of a population-wide expansion of invitation or the effect within the targeted group. The latter could be reflected by an ATT with respect to invitation.

Our primary estimand corresponds to the effect of invitation to DREAMS at the population level, reflecting a hypothetical intervention in which all AGYW in the population are invited early or later, compared to if all AGYW were not invited.

As such, the estimand is aligned with an intention-to-treat framework and captures the potential impact of a population-wide invitation( offering an intervention), rather than being restricted to the subgroup of AGYW who were actually invited. Furthermore, because a high proportion of AGYW were invited to DREAMS either early or later, the intervention became close to universal by 2022. In this context, we considered that estimating the average treatment effect (ATE) was more appropriate than estimating the average treatment effect among the treated (ATT) with the “treated” group in this case being DREAMS invitees.

We have clarified the definition of our estimand in page 12 line 307-311

• p. 13: Are there alternatives to IPTW that are not as sensitive to influential observations? If so, what is the benefit of using IPTW in this setting?

We acknowledge that inverse probability of treatment weighting (IPTW) can be sensitive to influential observations, particularly in the presence of extreme weights. Alternative approaches, such as outcome regression, propensity score methods (e.g., matching or stratification), may offer greater stability. However, these approaches also have limitations, such as reliance on correct model specification, potential loss of sample size (e.g. in propensity score matching), and susceptibility to residual confounding(e.g., in propensity score stratification).

We chose IPTW because it provides a framework for estimating the population-average causal effect under hypothetical intervention strategies, which aligns with our estimand of interest. It also allows for adjustment for time-varying confounding, and such adjustment was needed in our analyses because DREAMS invitation occurred both “early” (2016-2018) and “later” (2020-2022).

To mitigate sensitivity to influential observations, we used stabilised weights in the primary analysis and examined their distribution, and we found no evidence of extreme values. In addition, as a sensitivity analysis, we truncated stabilised weights at a value of 3 to assess the influence of extreme weights on the estimated effects (presented in supplementary material). The results were consistent with the primary analysis, suggesting that our findings are robust to the influence of extreme observations.

We have clarified the advantages of the use of the IPTW approach in page 28 line 591-595 that: *We used inverse probability of treatment weighting (IPTW) based on propensity scores to estimate the causal effect of DREAMS interventions on CMD while adjusting for time-dependent confounding and there were no missing data in the key confounding variables used in this analysis. We used stabilised weights and examined their distribution, and we found no evidence of extreme values.*

• p. 13, IPCW: Selecting variables based solely on their predictive ability for follow-up may not be sufficient to ensure appropriate adjustment for informative censoring. Please clarify the assumed censoring mechanism and the rationale for the variables included in the censoring model. A DAG could help make these assumptions more transparent.

Thank you for the feedback.

We agree that appropriate specification of the censoring model requires careful consideration of the underlying censoring mechanism, rather than relying solely on predictive ability. We assume that loss to follow-up in 2022 may be informative and driven by underlying socio-demographic and behavioural characteristics, such as age, invitation to DREAMS, pregnancy and sexual history selected apriori. These factors were also strong predictors of remaining under follow-up. We have clarified the assumed censoring mechanism in the Methods page 14 line 359-360; and we have clarified that the IPCW approach assumes that, conditional on these characteristics included in the censoring model, loss to follow-up is independent of the potential CMD outcomes. We have also included the censoring model in the supplementary material (S2 Table 4a and S2 Table 4b ).

• CMD prevalence among AGYW, by calendar year (Fig. 4 and Fig.5): There appears to be some variation in CMD prevalence across the early years of follow-up (2017–2019). The manuscript would benefit from a brief discussion of this pattern. Are there explanations for

this pattern, for example for the drop in CMD prevalence among later invitees in Nairobi between 2017 and 2019? Does the variation in CMD patterns prior to the implementation of the DREAMS program have any implications for the interpretation of the findings?

First, this decline may reflect secular trends in the external environment; for instance, the heightened socio-political instability in Kenya during the 2017 election cycle may have temporarily increased distress levels among AGYW, with subsequent stabilization by 2019 Second, the pattern may also reflect changes in the composition of DREAMS invitees over time. As the programme prioritised more vulnerable AGYW for early enrolment, individuals invited at later stages may represent a broader population. As such, the observed decline may reflect differences in the underlying characteristics of AGYW across calendar years rather than temporal changes in mental health.

• Could CMD levels measured in the early years of follow-up (2017–2019) be included in the adjustment set, if these measures are assumed to be associated with factors that influenced invitation?

We did not include CMD in the adjustment set for two reasons. First, CMD was not an eligibility criteria or assessment for invitation to DREAMS. Therefore, it was not a direct determinant of the invitation. Second, we excluded CMD due to its temporal relationship with the exposure. Because invitation to DREAMS occurred at different time points across individuals, CMD measured in 2017–2019 does not consistently represent a pre-exposure variable. For individuals invited early (2016–2018), CMD may have been measured after or during exposure, whereas for those invited later or not invited, it represents a pre-exposure characteristic.

As a result, CMD cannot be treated as a baseline confounder across all individuals, and adjusting for it could introduce bias through overadjustment. We therefore restricted the adjustment set to variables that were clearly pre-exposure for all individuals, as guided by the directed acyclic graph (DAG) (Figure 2).

• I agree that the COVID-19 pandemic may offer a possible explanation for the counterintuitive findings. Could differences in how individuals experienced and coped with the pandemic be related to life circumstances that also influenced invitation to the DREAMS program, and in turn affect CMD in 2022? If so, could this represent a source of residual confounding?

Thank you for the comment

We agree that differences in how individuals experienced and coped with the COVID-19 pandemic may be related to underlying socio-economic and behavioural factors, which could also have influenced both invitation to the DREAMS and subsequent mental health outcomes. As such, differential experiences of the pandemic could represent a potential source of residual confounding.

However, we expect that the impact of this to be limited. First, our analysis adjusts for a range of socio-economic and behavioural characteristics that are likely to capture key dimensions of vulnerability that also shaped both DREAMS invitation and COVID-19 pandemic-related experiences. Second, the COVID-19 pandemic affected the study population broadly, and while the magnitude of its impact may have varied, the direction of its effect on mental health is likely to have been similar across groups.

Additionally, the primary exposure of interest (invitation to DREAMS), was largely determined prior to or independently of pandemic-related experiences, and this reduces the likelihood that differential COVID-19 impacts substantially confounded the estimated effects. We have clarified this further in page 32 line 694-702.
