# Supplementary material for: Impact of the DREAMS interventions on Common mental disorders among adolescent girls and young women: Causal analysis of a prospective cohort in two Kenyan settings
Source: PLOS Ment Health. 2026 Nov 3;3(11):e0000765. doi: 10.1371/journal.pmen.0000765 (PMC13730345; doi:10.1371/journal.pmen.0000765)
Supplement: Peer review history

# Author comment: Author response to Decision Letter 0

**DOI:** 10.1371/journal.pmen.0000765.r001

3 Feb 2026

Attachment Submitted filename: response to reviewers.docx

# Peer review: Decision Letter 0

**DOI:** 10.1371/journal.pmen.0000765.r002

**Authors:** Andreas Haas

19 May 2026

PMEN-D-26-00057

Impact of the DREAMS interventions on Common mental disorders among adolescent girls and young women: Causal analysis of a prospective cohort in two Kenyan settings

PLOS Mental Health

Dear Dr. Magut,

Thank you for submitting your manuscript to PLOS Mental Health. After careful consideration, we feel that it has merit but does not fully meet PLOS Mental Health’s publication criteria as it currently stands. Therefore, we invite you to submit a revised version of the manuscript that addresses the points raised during the review process.

Please submit your revised manuscript by Jul 18 2026 11:59PM. If you will need more time than this to complete your revisions, please reply to this message or contact the journal office at mentalhealth@plos.org. When you're ready to submit your revision, log on to https://www.editorialmanager.com/pmen/ and select the 'Submissions Needing Revision' folder to locate your manuscript file.

Please include the following items when submitting your revised manuscript:

- A letter that responds to each point raised by the editor and reviewer(s). You should upload this letter as a separate file labeled 'Response to Reviewers'.
- A marked-up copy of your manuscript that highlights changes made to the original version. You should upload this as a separate file labeled 'Revised Manuscript with Track Changes'.
- An unmarked version of your revised paper without tracked changes. You should upload this as a separate file labeled 'Manuscript'.

Guidelines for resubmitting your figure files are available below the reviewer comments at the end of this letter.

As the corresponding author, your ORCID iD is verified in the submission system and will appear in the published article. PLOS supports the use of ORCID, and we encourage all coauthors to register for an ORCID iD and use it as well. Please encourage your coauthors to verify their ORCID iD within the submission system before final acceptance, as unverified ORCID iDs will not appear in the published article. *Only*  the individual author can complete the verification step; PLOS staff *cannot*  verify ORCID iDs on behalf of authors.

We look forward to receiving your revised manuscript.

Kind regards,

Andreas Haas

Academic Editor

PLOS Mental Health

**Journal Requirements:**

1. Please include a complete copy of PLOS’ questionnaire on inclusivity in global research in your revised manuscript. Our policy for research in this area aims to improve transparency in the reporting of research performed outside of researchers’ own country or community. The policy applies to researchers who have travelled to a different country to conduct research, research with Indigenous populations or their lands, and research on cultural artefacts. The questionnaire can also be requested at the journal’s discretion for any other submissions, even if these conditions are not met.  Please find more information on the policy and a link to download a blank copy of the questionnaire here: https://journals.plos.org/mentalhealth/s/best-practices-in-research-reporting. Please upload a completed version of your questionnaire as Supporting Information when you resubmit your manuscript.

2. Thank you for uploading your study's underlying data set. Unfortunately, the repository you have noted in your Data Availability statement does not qualify as an acceptable data repository according to PLOS's standards.

At this time, please upload the minimal data set necessary to replicate your study's findings to a stable, public repository (such as figshare or Dryad) and provide us with the relevant URLs, DOIs, or accession numbers that may be used to access these data. For a list of recommended repositories and additional information on PLOS standards for data deposition, please see https://journals.plos.org/plosone/s/recommended-repositories.

3. Please ensure that your Ethics Statement is available in its entirety at the beginning of your Methods section, under a subheading 'Ethics Statement'.

If the reviewer comments include a recommendation to cite specific previously published works, please review and evaluate these publications to determine whether they are relevant and should be cited. There is no requirement to cite these works unless the editor has indicated otherwise.

**Additional Editor Comments (if provided):**

The study addresses an important question and uses methods intended to support causal interpretation. I have concerns about the manuscript’s somewhat counterintuitive finding that the DREAMS intervention led to higher CMD symptoms. This finding requires careful scrutiny and explanation.

The reviewers considered the study to be generally well conducted. However, they identified several issues that require clarification and several methodological concerns that require substantial revision. In particular, the revised manuscript should address the following points:

1. The manuscript should provide a clearer and more detailed description of the eligibility criteria for the DREAMS intervention and the invitation process.

2. Please clarify the causal estimand.

3. The reviewers raised concerns about residual confounding. The revised manuscript should explain whether the available covariates adequately capture the factors that influenced DREAMS invitation. Baseline CMD levels may be especially relevant if they were associated with factors that influenced invitation. Please discuss whether baseline CMD can be included in the adjustment set and, if not, why not.

4. Please expand the discussion of the counterintuitive main finding in line with the reviewers’ recommendations.

5. Please provide more detail on censoring, the assumed censoring mechanism, and the use of inverse probability of censoring weights. Consider using a DAG.

6. Please clarify the intervention components that may affect mental health.

I invite you to submit a major revision. Please submit a point-by-point response to all reviewer comments and revise the manuscript accordingly. The revised manuscript will likely be returned to the reviewers for further assessment.

[Note: HTML markup is below. Please do not edit.]

Reviewers' comments:

Reviewer's Responses to Questions

**Comments to the Author**

1. Does this manuscript meet PLOS Mental Health’s publication criteria?

Reviewer #1: Yes

Reviewer #2: Yes

**********

2. Has the statistical analysis been performed appropriately and rigorously?

Reviewer #1: Yes

Reviewer #2: Yes

**********

3. Have the authors made all data underlying the findings in their manuscript fully available (please refer to the Data Availability Statement at the start of the manuscript PDF file)?

The PLOS Data policy

Reviewer #1: No

Reviewer #2: Yes

**********

4. Is the manuscript presented in an intelligible fashion and written in standard English?

Reviewer #1: Yes

Reviewer #2: Yes

**********

**Reviewer #1:**  The current manuscript evaluates the impact of *offering* the DREAMS interventions package on a secondary outcome, mental disorders. Overall, I find the paper to be well written and of decent methodological quality (and appropriate diagnostics). The critical part are the results and their interpretation (though I do not see any problem of reporting a “negative result”). The authors have offered some angles around that, but I suggest further disentangling in this regard. What could explain the results? I think there are at least the following options:

a) Measurement error in the outcome, due to the self-reporting of CMD. Discussed by the authors and speculated to be relevant.

b) An actual small negative effect. Appropriately discussed by the authors.

c) The ITT approach. Partly discussed. I agree that from a policy perspective “offering DREAMS” can be seen as a primary target. That’s fine. I still think there is no way around describing in more detail who, and how many participants, who were offered DREAMS accepted this. A potential explanation of the results is that there is a (small) benefit for the population who accepted DREAMS (only plausible pathway); and unmeasured residual confounding (see below) for the “vulnerable” group (with poorer outcomes) who did not. At a minimum the authors could describe proportion accepting the interventions and logistic models to describe who agreed to participate (at the different stages). A (encouraged) bonus would be using this information for reporting a short result on the effect of accepted DREAMS on the outcome (in the supplementary material), not to replace the initial analysis but to support the final interpretation of the primary results.

d) Unmeasured confounding. A strength of the study is the intense measuring of variables. However, treatment assignment/offering was based on the Girl Roster census method, whereas adjustment is based on “proxy variables”. I suggest the authors are very precise on the difference between them and explain to what degree they are different, to reflect on unmeasured confounding. Also, to what degree can one adjust for this “Girl Roster” method? Additionally, it would be helpful to see somewhere the logistic models for the probability of being offered (early/late) DREAMS, to understand who got it.

e) Time. It is CMD in 2022. Maybe there would be an effect if measured earlier, e.g shortly after the package was received. Can the authors comment on descriptive data being available for this and the potential impact?

f) Censoring. The authors used IPCW. The respective model could be reported, similar to the point raised under d). The possible censoring mechanism should be added to the DAG. Could there be unmeasured confounding with respect to drop-out?

g) Chance. Mayn hypothesized differences are consistent with data, given the assumptions. See also Sander Greenlands writings.

h) Assumptions violations. The reported diagnostics are however re-assuring in this respect.

My proposal is to work through these explanations, and do the above suggested small analyses, to then carefully re-visit conclusions and interpretations, as necessary.

As a side note: I find presenting results in Figures always nicer than in tables. The main results of the paper could be easily conveyed in that way.

**Reviewer #2:**  The manuscript is clearly structured and generally well written. My main concern relates to the findings, which appear somewhat counterintuitive. I suggest providing a more detailed description of the invitation process, including information on exactly who was eligible to be invited and how the selection criteria relate to the covariates included in the adjustment set.

I also recommend discussing baseline CMD levels across the observed comparison groups at the beginning of follow-up (2017–2019), and considering their inclusion in the adjustment set if appropriate. One might also reconsider the target estimand, for example by considering the average effect of invitation among those invited (i.e., an ATT with respect to invitation) as a complementary perspective. See detailed comments in the attached pdf.

**********

what does this mean?). If published, this will include your full peer review and any attached files.

**Do you want your identity to be public for this peer review?**  If you choose “no”, your identity will remain anonymous but your review may still be made public.

For information about this choice, including consent withdrawal, please see our Privacy Policy

Reviewer #1: No

Reviewer #2: No

**********

[NOTE: If reviewer comments were submitted as an attachment file, they will be attached to this email and accessible via the submission site. Please log into your account, locate the manuscript record, and check for the action link "View Attachments". If this link does not appear, there are no attachment files.]

**Figure Resubmissions:**

# Author comment: Author response to Decision Letter 1

**DOI:** 10.1371/journal.pmen.0000765.r003

27 Jul 2026

Attachment Submitted filename: Response to reviewers PMH.docx

# Peer review: Decision Letter 1

**DOI:** 10.1371/journal.pmen.0000765.r004

**Authors:** Karli Montague-Cardoso

8 Oct 2026

Impact of the DREAMS interventions on Common mental disorders among adolescent girls and young women: Causal analysis of a prospective cohort in two Kenyan settings

PMEN-D-26-00057R1

Dear Magut,

We are pleased to inform you that your manuscript 'Impact of the DREAMS interventions on Common mental disorders among adolescent girls and young women: Causal analysis of a prospective cohort in two Kenyan settings' has been provisionally accepted for publication in PLOS Mental Health.

Before your manuscript can be formally accepted you will need to complete some formatting changes, which you will receive in a follow up email. A member of our team will be in touch with a set of requests.

Please note that your manuscript will not be scheduled for publication until you have made the required changes, so a swift response is appreciated.

IMPORTANT: The editorial review process is now complete. PLOS will only permit corrections to spelling, formatting or significant scientific errors from this point onwards. Requests for major changes, or any which affect the scientific understanding of your work, will cause delays to the publication date of your manuscript.

If your institution or institutions have a press office, please notify them about your upcoming paper to help maximize its impact. If they'll be preparing press materials, please inform our press team as soon as possible -- no later than 48 hours after receiving the formal acceptance. Your manuscript will remain under strict press embargo until 2 pm Eastern Time on the date of publication. For more information, please contact mentalhealth@plos.org.

Thank you again for supporting Open Access publishing; we are looking forward to publishing your work in PLOS Mental Health.

Best regards,

Karli Montague-Cardoso

Staff Editor

PLOS Mental Health

***********************************************************

Reviewer Comments (if any, and for reference):

Reviewer's Responses to Questions

**Comments to the Author**

Reviewer #2: All comments have been addressed

**********

publication criteria?

Reviewer #2: Yes

**********

3. Has the statistical analysis been performed appropriately and rigorously?

Reviewer #2: Yes

**********

4. Have the authors made all data underlying the findings in their manuscript fully available (please refer to the Data Availability Statement at the start of the manuscript PDF file)?

The PLOS Data policy

Reviewer #2: Yes

**********

5. Is the manuscript presented in an intelligible fashion and written in standard English?

Reviewer #2: Yes

**********

Reviewer #2: (No Response)

**********

what does this mean?). If published, this will include your full peer review and any attached files.

**Do you want your identity to be public for this peer review?**  If you choose “no”, your identity will remain anonymous but your review may still be made public.

For information about this choice, including consent withdrawal, please see our Privacy Policy

Reviewer #2: No

**********
